# Transcriptomics analysis identifies folding and secretion related genes for improving monoclonal antibody production in *Thermothelomyces heterothallica* C1

**DOI:** 10.1186/s12934-026-02989-w

**Published:** 2026-04-02

**Authors:** Mari Mäkinen, Antti Aalto, Tiina Pakula, Marilyn G. Wiebe, Anne Huuskonen, Marika Vitikainen, Mari Valkonen, Sami Havukainen, Ellinor Englund, Veera Korja, Mark Emalfarb, Noelia Valbuena Crespo, Ronen Tchelet, Markku Saloheimo

**Affiliations:** 1 VTT Technical Research Centre of Finland Ltd., P.O. Box 1000, Espoo, 02044 VTT Finland; 2 Onego Bio Ltd., Hämeentie 157, Helsinki, 00560 Finland; 3 Thermo Fisher Scientific Oy, Ratastie 2, Vantaa, 01620 Finland; 4 Dyadic Applied BioSolutions, 1044 North U.S. Highway One, Suite 201, Jupiter, FL 33477- 5094 USA

**Keywords:** *Thermothelomyces heterothallica*, Secretory pathway, Monoclonal antibody production, Secretion stress, Glycoengineering, Protein folding

## Abstract

**Background:**

The filamentous fungus *Thermothelomyces heterothallica* C1 has been developed into a highly productive protein production system for heterologous proteins like antibodies and vaccine candidates. While it is capable of secreting over 120 g/l of its native enzymes, monoclonal antibodies (mAbs) have been produced at titers exceeding 20 g/l, and strains engineered to produce human-type N-glycan structures have been developed. However, significant variability in mAb productivity and reduced production levels in glycoengineered strains limit the use of C1 as a widespread production host in the pharmaceutical industry. To address these issues, transcriptome analysis was conducted on strains producing five different mAbs with varying production efficiencies, as well as on mAb-producing strains with native and glycoengineered N-glycans. Genes related to protein folding and secretion, which are regulated in response to mAb production and glycoengineering, were over-expressed in a glycoengineered mAb producing C1 strain. In addition, genes identified based on previously described functions in the secretory pathway, including counterparts of human origin, were included in the study.

**Results:**

Transcriptome analysis revealed that the mAb heavy and light chains were among the most abundantly expressed transcripts, indicating that production bottlenecks occur after transcription. Genes associated with protein folding, quality control, glycosylation, and transport within the secretory pathway were upregulated in the mAb-producing strains. This upregulation was more pronounced in strains with low mAb yields and in glycoengineered strains. The over-expression of 10 genes (*bet1*, *dnaj*-type gene, *dpm1*, *ero1*, *erv46*, human calreticulin, human *cypb*, human *mzb1*, *pmr1*, and UDP-galactose transporter), each playing distinct roles in the secretory pathway, enhanced mAb production in glycoengineered C1 strains from 1.5- to 2.5-fold.

**Conclusions:**

Through a comprehensive analysis of transcriptome data from C1 strains producing various monoclonal antibodies (mAbs) and an extensive literature search, several factors related to protein folding and secretion were identified as potential targets for enhancing mAb production. The over-expression of some of these genes in glycoengineered C1 strains led to improvement in mAb production, with some genes enhancing mAb yields by 2.5-fold.

**Supplementary Information:**

The online version contains supplementary material available at 10.1186/s12934-026-02989-w.

## Background

The filamentous fungus *Thermothelomyces heterothallica* C1 is a thermotolerant haploid ascomycetous fungus which was originally known for its ability to secrete high amounts of biomass-hydrolyzing enzymes [1]. *T. heterothallica* has undergone extensive random mutagenesis and genetic engineering to enhance its protein production capabilities, with the aim of developing a highly productive expression system capable of secreting large amounts of heterologous proteins, such as immunologically active antigens and antibodies [2–6]. Unlike bacterial hosts for heterologous protein production, C1 can perform eukaryotic post-translational modifications, which are crucial for the proper folding and function of proteins. Compared to mammalian cells as production hosts, cultivating C1 is faster, more economical and straightforward, and genetic manipulation is easier. One of the benefits of the current C1 system as a production host for heterologous proteins is the availability of glycoengineered strains with mammalian-type N-glycosylation [7, 8]. The native Man3-Man9 type glycans incorporated by C1 into secreted proteins are less complex and more human-like than the high-mannose type glycans produced by yeast [9, 10]. Through genetic engineering, C1 strains producing proteins with G0 (no galactoses attached to the arms of biantennary glycans) and G1 and G2 (one or two galactoses attached to the arms of biantennary glycans) types of glycans have been constructed (our unpublished data).

Some challenges remain in the production of monoclonal antibodies (mAbs) in C1. It is not well understood why some mAbs are produced at higher levels than others (our unpublished data). Additionally, production levels in glycomodified C1 strains are consistently lower than in strains that incorporate Man3-Man9 type fungal N-glycans, and the mechanisms behind this phenomenon are unknown (our unpublished data). Monoclonal antibodies are glycoproteins containing two identical heavy chains (HC) and two identical light chains (LC). The complex tertiary structures, multiple disulfide bridges, and large size of mAbs likely create a burden on the folding and secretory machinery of the fungus.

Efficient protein secretion relies on proper protein processing and quality control mechanisms, including correct folding and glycosylation of secreted proteins. Additionally, the removal of incorrectly folded proteins is managed by endoplasmic reticulum-associated degradation (ERAD) and the unfolded protein response (UPR) [11]. The secretion process in eukaryotic cells begins with the translocation of proteins into the lumen of the endoplasmic reticulum (ER), where several modifications occur [12]. These include signal sequence cleavage, chaperone-assisted folding, disulfide bond formation and glycosylation, all of which ensure that proteins are properly folded and functional. Among the molecular chaperones of the heat shock protein family (Hsp), the Hsp70 family chaperone BIP (binding immunoglobulin protein) is the most abundant in the ER [13]. Heterologous protein production is often associated with increased levels of chaperones that promote the correct folding of the unfolded polypeptides [12]. Disulfide bond formation, a post-translational modification essential for protein stability and biological activity, is catalyzed by the protein disulfide isomerase that also functions as a chaperone by preventing peptide misfolding [14]. Initial glycosylation of secreted proteins occurs in the ER, where a cascade of different enzymes adds sugars onto a lipid carrier to form the core oligosaccharide chain that is transferred to the polypeptide [15]. After the protein enters a Golgi-like structure, further modifications of the *N*-glycans take place. Glycosylation capacity of the secretory pathway may influence the efficiency of heterologous protein production.

The unfolded protein response is activated when large amounts of unfolded or misfolded proteins accumulate in the ER, causing stress, or when the ER’s capacity is exceeded by high protein production [16, 17]. Consequently, the expression of folding-related genes is induced by the spliced form of *hac1*, and proteins are targeted to the ERAD machinery [18, 19]. This mechanism may limit production of heterologous proteins, as they can be recognized as aberrant, or the ER’s capacity might be exceeded due to the over-expression of the corresponding heterologous gene.

Efficient secretion also requires effective vesicle trafficking between the endoplasmic reticulum and the Golgi apparatus, and finally to the plasma membrane and extracellular matrix [20, 21]. Properly folded proteins are packaged into coat protein complex II (COPII) transport vesicles, in which the proteins are trafficked through the Golgi complex and eventually to secretory vesicles. These secretory vesicles then fuse with the plasma membrane to release the proteins. Vacuole-targeted proteins remain as intracellular proteins or undergo proteolytic degradation.

In this study, five different monoclonal antibodies, each displaying varying production efficiencies, were expressed in C1 with either native glycans or glycomodifications. The generated transcriptome data and literature were utilized to select genes involved in various steps of protein folding, processing, and export that were overexpressed in an antibody-producing strain to address the effect of their overexpression on mAb productivity.

## Methods

### Strain construction

C1 strains producing Keytruda, Humira, Nivolumab, mAb1 and mAb2 were constructed by expressing the mAbs under the native *bgl8* promoter from *cbh1* locus. Nivolumab was additionally expressed in the similar manner in three glycomodified parental strains and Humira was expressed also under the synthetic AnSES promoter from the *cbh1* locus (Table 1).

To produce the different mAbs in the C1 expression system, heavy chain (HC) and light chain (LC) sequences were first codon-optimized and synthesized by a gene synthesis company (Genscript, USA). The native cellobiohydrolase 1 (CBH1) signal sequence was added to the N-terminus of both chains and sequences overlapping with the C1 expression vectors were added to both ends of the HC and LC fragments.

The HC and LC genes were released from the Genscript vectors by MssI digestion or amplified by PCR and cloned into the PacI site of the C1 expression vector pMYT0070 and pMYT0069, respectively, by Gibson assembly (HiFi DNA Assembly Cloning Kit, New England Biolabs, USA). Correct sequences of the resulting expression vectors were verified by DNA sequencing. Expression constructs included the C1 *bgl8* promoter for both HC and LC. Humira was also produced under a synthetic AnSES promoter [22]. The terminator from a native chitinase gene (chi1t) was selected for HC genes and *bgl8* terminator for LC genes. All constructs were directed to the *cbh1* locus via homologous targeting sequences from the 5´ and 3´ flanking regions. Constructs were designed as split vectors which means that the hygromycin marker was separated in two different overlapping fragments and during transformation these fragments recombined to form full length, functional selection marker. This resulted in integration of the HC and LC expression cassettes in opposite orientations.

Fragments containing the expression cassette, overlapping fragments of the selection marker, and the 5′ and 3′ flanking sequences for integration to the *cbh1* locus were released from the HC and LC expression plasmids with MssI digestion and co-transformed into the C1 production strain DNL125 from which 8 protease genes (*alp1*, *alp2*, *pep4*, *prt1*, *srp1*, *alp3*, *pep1* and *mtp2*) had been deleted. In addition, Nivolumab was transformed to Δalg3, G0 and G2 strains (M2897, M3563, M3693). ALG3 is an alpha-1,3-mannosyltransferase that catalyzes the addition of the first dol-P-Man derived mannose to Man5GlcNAc2-PP-Dol. In the G0 and G2 strains, the glycomodification cassettes, expressing human GNT1, rat GNT2 (glucose N-acetyltransferases) and human galactosyltransferase GalT (only for G2 strain) [7], had been integrated to the *alp6* locus. In the G0 and G2 strains, the *alg11* gene had been deleted. ALG11 is an alpha-1,2-mannosyltransferase that catalyzes the transfer of the fourth and fifth mannose residues from GDP-mannose to Man3GlcNAc2-PP-dolichol and Man4GlcNAc2-PP-dolichol. N-glycans of the strains had been analyzed as described previously [4] and the results are shown in Additional file 1., Supplementary Fig. 6. Transformation was done as described previously [7].

Transformants were selected for hygromycin resistance and streaked on selective medium plates. The transformants were screened for mAb production by growing them in 24-well plates (35 mM (NH_4_)_2_SO_4_, 7 mM NaCl, 55 mM KH_2_PO_4_, 0.1% yeast extract, 0.5% glucose, 2 mM MgSO_4_, 1 x trace elements, 10 mM uridine, pH 6.5) and performing Western immunoblotting on the culture supernatants as described previously [3]. Gel electrophoresis was performed in 18-well or 26-well Criterion TGX Precast 4–20% Gels (BioRad, USA). For HC detection, anti-human IgG F(c) Goat Polyclonal Antibody DyLight680 (Li-Cor, USA) was used. For kappa LC detection, Goat anti-human Kappa Light Chain secondary antibody DyLight 800 (Li-Cor, USA) was used. For lambda LC detection, anti-human lambda light chain antibody (Abcam, UK) was used together with goat anti-mouse 800CW secondary antibody. Transformants producing the different mAbs were purified through single colony cultures and analyzed by PCR for correct integration into the *cbh1* locus and the absence of the *cbh1* coding region, using oligonucleotide primers listed in Additional file 1., Supplementary Table 1. Correct 5´ and 3´ integration of the expression cassettes to the *cbh1* locus was demonstrated by amplifying 2.3 kb 5’ end and 1 kb 3 ’end fragments from the transformants. Purity of the clones was verified by loss of an internal 0.5 kb *cbh1* gene fragment. The production strains used in further analyses were selected based on Western blot and PCR screening results and stored as master cell banks (MCB) at -80 °C.

### Bioreactor cultivations and sample analyses

The selected production strains were cultivated in a Sartorius Ambr^®^ 250 High Throughput -system (Sartorius, Germany) in three replicate reactors. Cultures were maintained at pH 6.4 ± 0.1, 38 °C, in a fed-batch process for 6 days, as described in [3]. The feed was started automatically when carbon dioxide concentration began to decrease, indicating that all glucose in the batch phase had been consumed.

The first cultivation group included three biological replicates of the non-glycomodified antibody producing strains (mAb2, Keytruda, Humira under bgl8p and AnSESp) together with the DNL125 parental strain. The second group of cultivations included the mAb1 producing strain and all Nivolumab producing strains, together with the parental strain. Samples were collected daily from the cultivations. Protease inhibitor Phenylmethanesulfonyl fluoride (PMSF) was added immediately to all samples prior to centrifugation at a final concentration of approximately 1.5 mM. The samples were centrifuged at 13 000 rpm for 5 min, with and without addition of an equal volume of buffer, to separate mycelia from the supernatant. Supernatants from samples without added buffer were collected for affinity purification and western blot analyses and the pellets were used for dry weight analysis. Mycelial pellets which had been washed by adding buffer were collected for RNA extraction, the diluted supernatant was discarded and the pellets immediately frozen in liquid nitrogen for subsequent storage at -80 °C. In total, 66 mycelium samples were collected for RNA extraction and purification from three biological replicate cultures on days 2 and 4 of the bioreactor cultivations.

Secretion of the different mAbs was confirmed and quality of the mAbs during bioreactor cultivations was estimated with Western immunoblotting on the culture supernatants as described previously [3].

The different mAbs were purified chromatographically from the 6-day culture supernatants with a 1 ml MabSelect PrismA or MabSelect SuRe protein A resin (Cytiva, USA) on an ÄKTA Start automated HPLC system (GE Healthcare, UK) according to manufacturer´s protocols. The purified mAb was quantified based on absorbance at 280 nm by integrating the elution peak with the Unicorn 7 software (Cytiva, USA).

### RNA extraction

Total RNA was isolated using TRIzol reagent (Thermo Fisher Scientific, USA). 1 ml of Trizol was added to the frozen mycelium sample and mixed by pipetting up and down. Sample was transferred to a microcentrifuge tube containing 0.5 ml glass beads and cells were disrupted with a homogenizer (Precellys, Bertin Technologies, France). The first homogenization cycle was 5500 rpm 2 × 30 s and the second 6500 rpm 1 × 30 s, after which 0.2 ml of chloroform was added and samples were centrifuged 12 000 g for 15 min at + 4 °C. The clear upper phase was transferred to a new microcentrifuge tube, 0.5 ml of isopropanol was added and the sample was centrifuged at 12 000 g for 8 min at room temperature. Supernatant was removed, the pellet was washed with 75% (v/v) ethanol and centrifuged at 7500 g for 5 min at + 4 °C. Supernatant was removed, the pellet was dried and resuspended in 50 µl of RNase free water. RNA was purified using an RNeasy Mini Kit (Qiagen, Germany) and RNA concentration was measured using NanoDrop ND-1000 (NanoDrop Technologies Inc., USA). Integrity of the RNA was analyzed using an Agilent 2100 Bioanalyzer (Agilent Technologies, USA).

### RNA-seq data analysis

RNA extracted from samples of the triplicate cultures of each strain at two times were sequenced at Source BioScience (Cambridge, UK). Stranded sequencing produced 150 bp pair-end reads. The quality of the reads was assessed with FastQC quality control software [23] before and after trimming with Fastp [24]. Reads were aligned with HISAT2 [25]. Stringtie [26] was used for transcript assembly and quantification. Differential expression of genes was analyzed with DESeq2 [27] using *p* < 0.01 and log2 fold change > 1 or <-1 as the threshold. The R package Mfuzz was used for soft clustering of genes [28]. Venn diagrams were drawn in R using ggVennDiagram library [29].

### Over-expression of selected genes in glycomodified mAb producing C1 strain

Genes selected from the transcriptome data and based on a literature search were cloned between the native C1 TEF1A promoter and a yeast ADH1 terminator in an expression cassette that was targeted to the *alp6* protease locus via homologous targeting sequences from the 5´ and 3´ flanking regions. Transformants were selected based on the *pyr4* selection marker. Expression cassettes were released from the vector backbone by MssI digestion and transformed to C1 strain M4575 in which 8 protease genes (*alp1*, *alp2*, *pep4*, *prt1*, *srp1*, *alp3*, *pep1* and *mtp2*) had been deleted, G2 glycosylation machinery had been incorporated, and Nivolumab was expressed under the *bgl8* promoter from the *cbh1* locus. Glycomodification of this strain was carried out previously by first deleting *alg3* with a cassette including *pyr4* selection, after which the *pyr4* marker was looped out and Nivolumab expression cassette was added with nia1-hygr selection. Finally, a glycomodification cassette was targeted to the *alg11* locus using amdS selection. N-glycans of the strain had been analyzed as described previously [4] and the results are shown in Additional file 1., Supplementary Fig. 6.

Transformants growing as colonies were selected based on their ability to grow without uridine/uracil and streaked on selective medium plates. Transformants were screened for Nivolumab production by growing them in 24-well plates and performing Western immunoblotting on the culture supernatants as described previously [3]. Transformants were purified through single colony cultures and analyzed by PCR for correct integration into the *alp6* locus and the absence of the *alp6* coding region using oligonucleotide primers listed in Additional file 1., Supplementary Table 1. Correct 5´ and 3´ integration of the expression cassettes to the *alp6* locus was demonstrated by amplifying 1.7 kb and 1.6 kb fragments, respectively. Purity of the clones was verified by loss of internal 0.9 kb fragment of the *alp6* gene.

### Bioreactor cultivations of strains over-expressing different folding and secretion related factors and sample analyses

Selected strains were cultivated in Sartorius Ambr^®^ 250 High Throughput -system (Sartorius, Germany) for 7 days.

Production level of Nivolumab during bioreactor cultivations of the different over-expression strains was first estimated with Western immunoblotting on the culture supernatants as described previously [3]. Nivolumab was purified from the 7-day culture supernatants by protein A chromatography with a 1 ml MabSelect PrismA or MabSelect SuRe column (Cytiva, USA) on an ÄKTA Start automated HPLC system (GE Healthcare, UK) according to the manufacturer´s protocols and quantified as described above for production of the different mAbs. The quality of the purified Nivolumab was assessed for both reduced (with β-mercaptoethanol) and non-reduced (without β-mercaptoethanol and without heating) samples by Western blot and stained SDS PAGE analyses. SDS PAGE gels were stained with PageBlue protein staining solution (Thermo Fisher Scientific, USA).

## Results

### Production of different monoclonal antibodies in C1

To investigate the production of five different monoclonal antibodies (mAbs) by C1, codon-optimized genes encoding the heavy chains (HC) and light chains (LC) were synthetized, cloned to C1 compatible expression vectors and integrated into the *cbh1* locus of a native glycan producing C1 strain (Table 1). These mAbs were chosen based on prior studies that showed varying production levels and stabilities in C1 (our unpublished data). They included mAbs already in use as prescription medicines, pembrolizumab (Keytruda), adalimumab (Humira) and nivolumab (Opdivo), and mAb1 and mAb2 that are proprietary antibodies of our collaborators. To enable comparison between the production strains, all mAbs were expressed similarly under the native *bgl8* promoter from the same locus (*cbh1*). Additionally, Nivolumab was expressed in three glycomodified parental strains, and Humira was expressed under the synthetic AnSES promoter [22] that has shown significantly higher expression levels than the *bgl8* promoter (data not shown). The glycoengineered strains had been modified by the deletion of the *alg3* gene and/or contained alterations towards G0 and G2 glycosylation types (Additional file 1., Supplementary Fig. 6). Both *alg3* and *alg11* were deleted in G0 and G2 type strains. These strains also contained expression constructs for GlcNAc transferases GNT1 and GNT2. The G2 strain had additionally an expression cassette for the galactosyltransferase GalT.

Transformants were screened for mAb production with 24-well cultures and Western blot analyses. The identified production strains were purified through single colony cultures and shown by PCR to have the correct integration of the mAb expression cassette to the *cbh1* locus. Nine constructed strains were cultivated in bioreactors in three replicate cultures alongside the parental strain. Supernatant samples from the cultivations were analyzed with Western blots (Additional file 1., Supplementary Fig. 1). All constructed strains produced mAbs throughout the cultivation, with production levels increasing towards the end. In many cases, the light chain (LC) was produced more efficiently than the heavy chain (HC), particularly in the cases of Humira under AnSESp and in Keytruda.

**Table 1:** Monoclonal antibodies produced in C1 strains with native glycans or with glycomodifications

mAb produced	Promoter	Locus	Parental strain background
Nivolumab	bgl8	*cbh1*	Native glycans
Nivolumab	bgl8	*cbh1*	Δ*alg3*
Nivolumab	bgl8	*cbh1*	G0
Nivolumab	bgl8	*cbh1*	G2
mAb1	bgl8	*cbh1*	Native glycans
mAb2	bgl8	*cbh1*	Native glycans
Humira	bgl8	*cbh1*	Native glycans
Humira	AnSES	*cbh1*	Native glycans
Keytruda	bgl8	*cbh1*	Native glycans

**Fig. 1:**
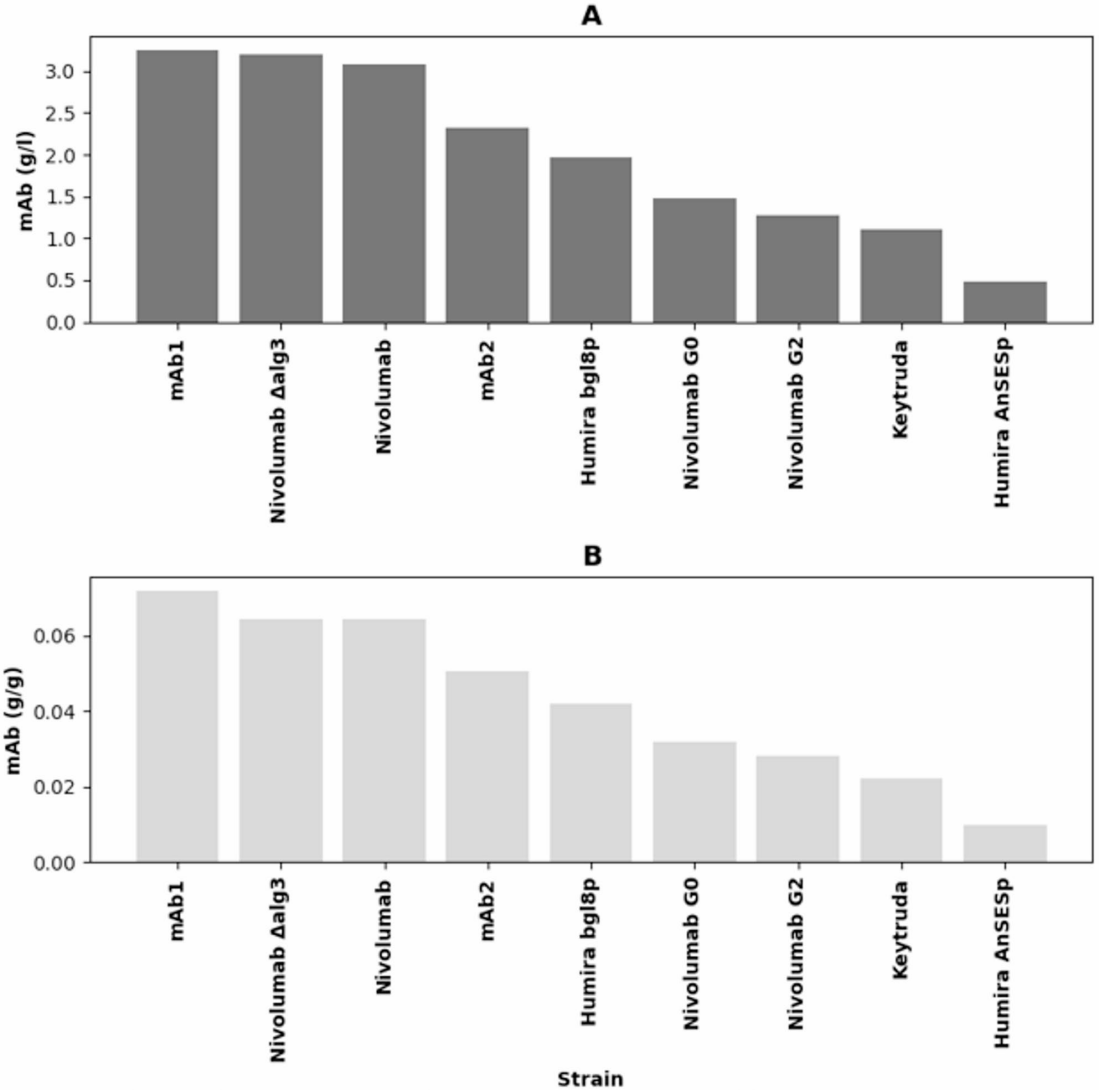
Production levels of different monoclonal antibodies at 6-day time point of bioreactor cultivations. **A** shows the volumetric production level and in **B** the production level has been normalized against the biomass dry weight of the cultivation. Production levels were determined for one replicate bioreactor cultivation

Quantification by protein A affinity chromatography purification of the different mAbs from the 6-day time point of bioreactor cultivation indicated that mAb1 and Nivolumab were produced at the highest levels, whereas Keytruda and Humira under the AnSES promoter were produced at the lowest production levels (Fig. 1A). Strains producing Humira under the bgl8p and mAb2 had moderate productivity. Deletion of the *alg3* gene did not compromise Nivolumab production, whereas glycomodifications towards G0 and G2 types of glycans clearly reduced the production. Glycomodifications did not have a negative effect on biomass production during the bioreactor cultivations (Fig. 1B).

### Transcriptome analysis by RNA-sequencing

**Fig. 2:**
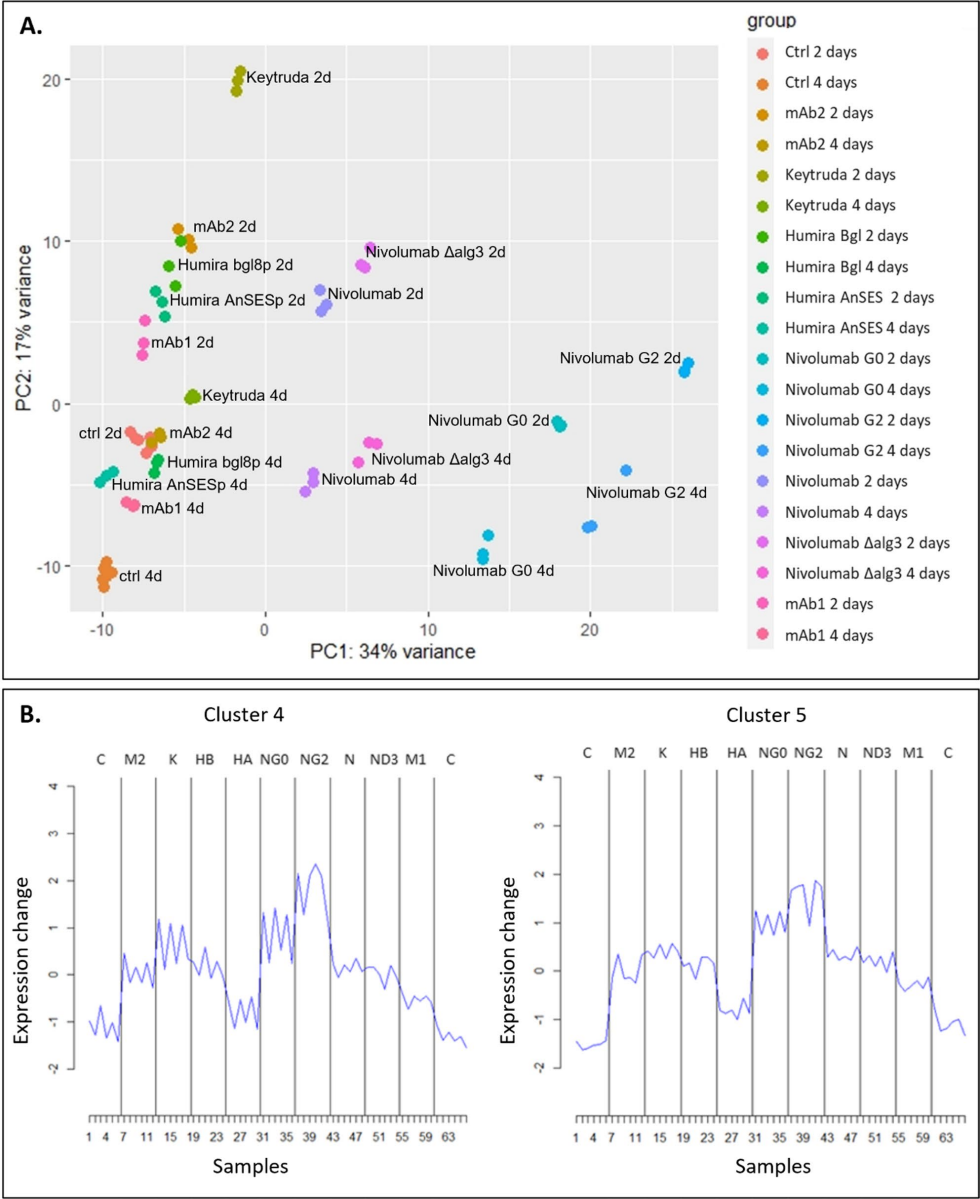
Principal component analysis of the RNA-sequencing samples from 2-day and 4-day bioreactor cultivations (**A**) and gene expression profiles for cluster centers of clusters 4 and 5 (**B**). In **B**, biological replicates and cultivation timepoints are shown separately. For example, samples 1–6 correspond to control strain replicate 1 day 2, replicate 1 day 4, replicate 2 day 2, replicate 2 day 4, replicate 3 day 2 and replicate 3 day 4. Black lines separate the mAb producing strains and control strains from each other. C/Ctrl: control, M2: mAb2, K: Keytruda, HB: Humira under bgl8p, HA: Humira under AnSESp, NG0: Nivolumab in G0 strain, NG2: Nivolumab in G2 strain, N: Nivolumab in strain with native glycans, ND3: Nivolumab in alg3 deletion strain, M1: mAb1

The transcriptomes of three biological replicate cultures of the mAb production strains and the parental strain were analyzed at two times (2 days and 4 days) during bioreactor cultivations, using RNA sequencing (RNA-seq). This analysis aimed to identify gene expression changes that might reflect the varying production levels of different mAbs. Differences in gene expression were examined between the antibody-producing strains and the parental strain, as well as between glycomodified and non-glycomodified strains. Principal component analysis (PCA) indicates the cultivation time and glycomodification of the strains as the major sources of variation (Fig. 2A). Keytruda 2-day samples differed clearly from other samples of the same time. Similarly, 2-day samples of non-glycomodified Nivolumab and Δalg3 Nivolumab were distinct from other mAbs. 2-day samples of G0 and G2 Nivolumab cultivations did not group with the 2-day samples of other mAbs but were closer to 4-day samples of glycomodified and non-glycomodified Nivolumab cultivations. Δalg3 Nivolumab samples were more similar to non-glycomodified Nivolumab samples of the same time than to G0 and G2 samples, indicating that glycomodifications towards G0 and G2 types of glycans cause distinct changes to gene expression.

Soft clustering with Mfuzz package was carried out to study the different expression profiles present in the data and to identify genes with similar profiles. Genes were separated into 20 clusters. Differential expression analysis identified approximately 1,500 genes that exhibited significant differences in expression across all the comparisons studied (*p* < 0.01, log2 fold change > 1 or <-1). Genes that had very low counts in all samples were filtered from the data. From Mfuzz clustering analysis of the transcription data (Additional file 1., Supplementary Fig. 2), two clusters (clusters 4 and 5) were identified that contained genes which were up-regulated in several antibody-producing strains (Fig. 2B). From the transcriptome data, 129 and 59 genes were assigned to clusters 4 and 5, respectively. These clusters were utilized later in the study to select genes with specific expression profiles for over-expression experiments whereas here we will address all the genes that were differentially expressed.

The highest number of differentially expressed genes was found in the glycomodified Nivolumab strains and the strain producing the Keytruda mAb (Table 2). Conversely, the lowest number of differentially expressed genes was observed in the mAb1-producing strain and the strain producing Humira under the AnSES promoter. In most samples, the number of differentially expressed genes was lower at day 4 than at day 2.

**Table 2:** Number of differentially expressed genes in the mAb producing strains during bioreactor cultivations

Produced mAb	Compared to strain	Up-regulated genes, 2 days	Up-regulated genes, 4 days	Down-regulated genes, 2 days	Down-regulated genes, 4 days
mAb1	Parental (native glycans)	19	16	5	2
mAb2	Parental (native glycans)	43	42	21	11
Humira bgl8p	Parental (native glycans)	57	50	14	18
Humira AnSESp	Parental (native glycans)	28	8	8	5
Keytruda	Parental (native glycans)	311	92	205	37
Nivolumab	Parental (native glycans)	67	58	17	17
Nivolumab Δalg3	Parental (native glycans)	199	98	89	59
Nivolumab G0	Parental (native glycans)	170	136	152	88
Nivolumab G2	Parental (native glycans)	463	284	479	221
Nivolumab Δalg3	Nivolumab (native glycans)	53	16	27	29
Nivolumab G0	Nivolumab (native glycans)	58	36	145	45
Nivolumab G2	Nivolumab (native glycans)	243	85	237	130

**Fig. 3:**
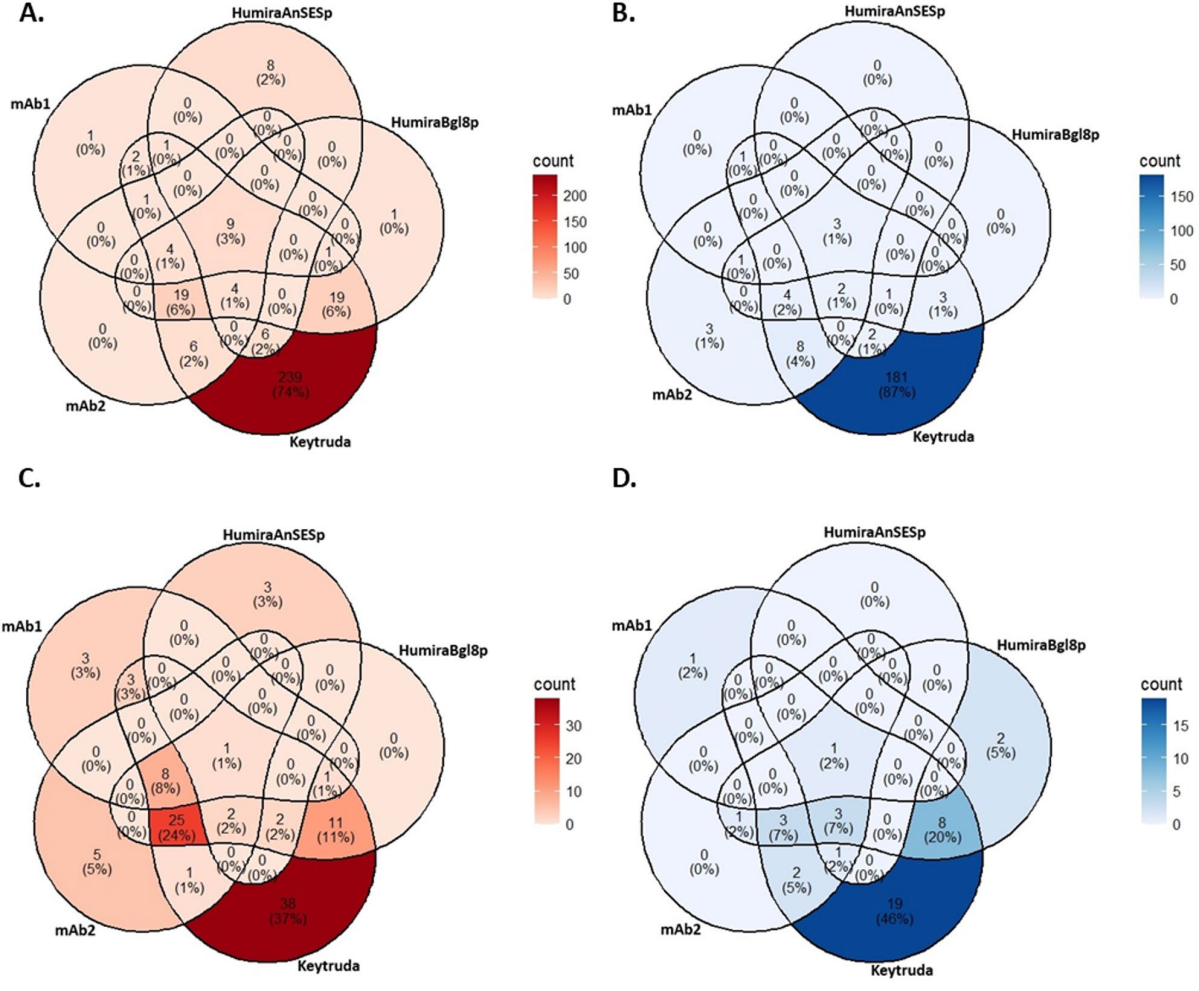
Venn diagrams of genes up-regulated or down-regulated in strains producing different mAbs. Number of genes up-regulated (**A** and **C**; p-value < 0.01 and log2 fold change > 1) or down-regulated (**B** and **D**; p-value < 0.01 and log2 fold change < -1) at day 2 (**A** and **B**) and day 4 (**C** and **D**) of the bioreactor cultivations compared to the empty parental strain (DNL125)

C1 strain producing Keytruda differed from the other strains by regulating large numbers of genes that were not up- or down-regulated in other mAb producing strains (Fig. 3). The uniquely up-regulated genes included putative annotations such as proteins needed for the translocation across the ER membrane (*Sec61*, *Sec62 *and *Sec63*), subunits from signal peptidase, dolichol-diphosphooligosaccharide protein glycosyltransferase (ost), dolichol-phosphate mannosyltransferase (dpm) and oligosaccharyltransferase (stt3) complexes, ER membrane proteins and E3 ubiquitin ligases involved in ERAD pathway, proteins involved in ER exit of secreted proteins, ER resident peptidyl-prolyl isomerase (PPIase), stress response protein Rds1, Golgi membrane protein tpv23, vacuolar sorting receptor, ER heat shock proteins (*dnaJ*, *scj1*) and a dolichyl phosphate N-acetylglucosaminephosphotransferase (*alg7*) involved in protein glycosylation.

**Fig. 4:**
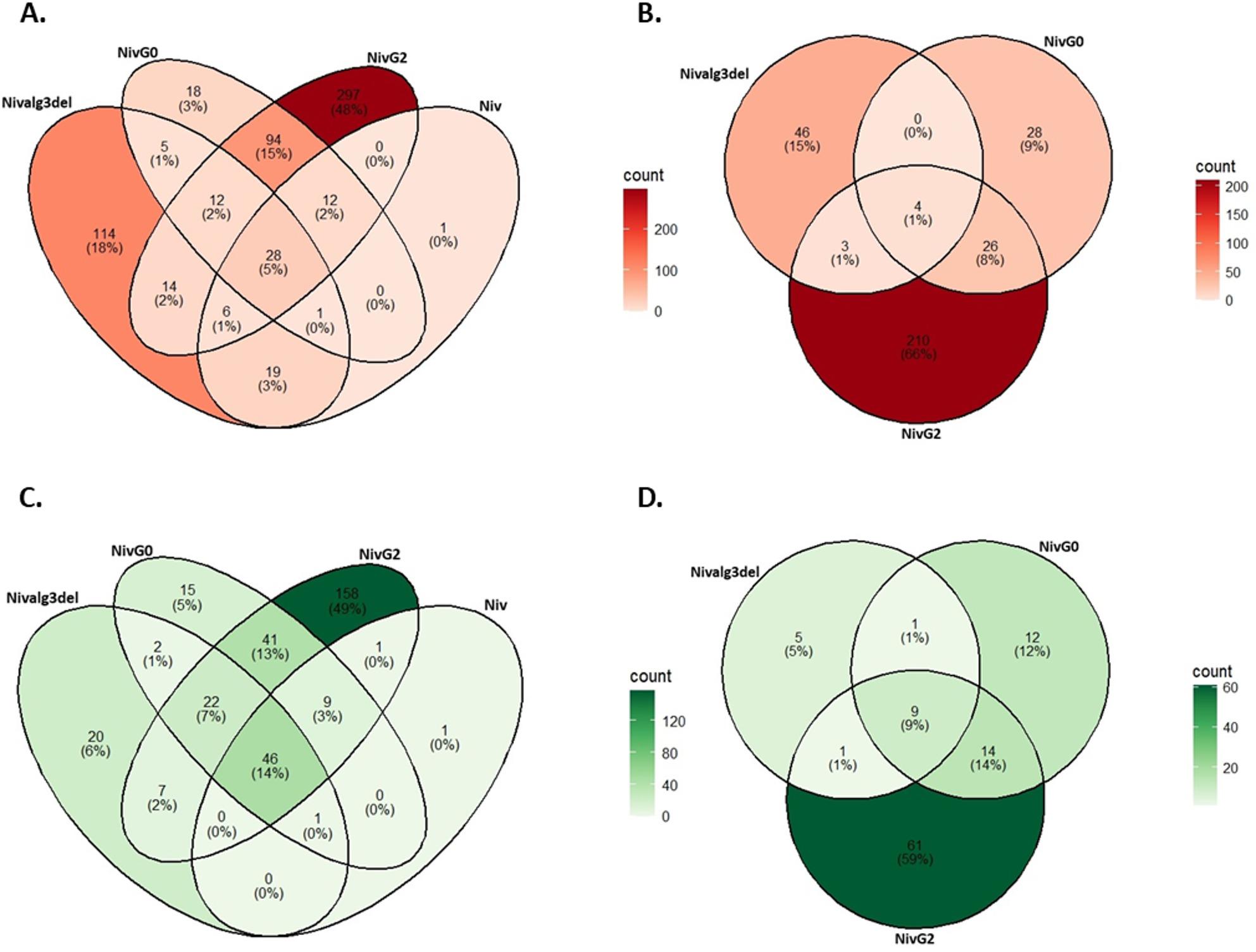
Venn-diagram of genes up-regulated in glycomodified and non-glycomodified Nivolumab producing strains. Number of up-regulated (p-value < 0.01 and log2 fold change > 1) genes at days 2 (A and B) and 4 (C and D) of the bioreactor cultivations. Fold change was calculated against the samples of the empty parental strain (DNL125, A and C) or against the Nivolumab producing strain with native glycans (B and D). Niv: Nivolumab, alg3del: alg3 deletion

When comparing glycomodified Nivolumab production strains to the non-mAb-producing parental strain with native glycans, the number of differentially expressed genes was higher than when comparing to the Nivolumab strain with native glycans (Table 2; Fig. 4). This suggests that mAb production has a stronger influence on gene expression than glycomodification. The largest number of genes were upregulated in G2 Nivolumab strains (Fig. 4).

**Fig. 5:**
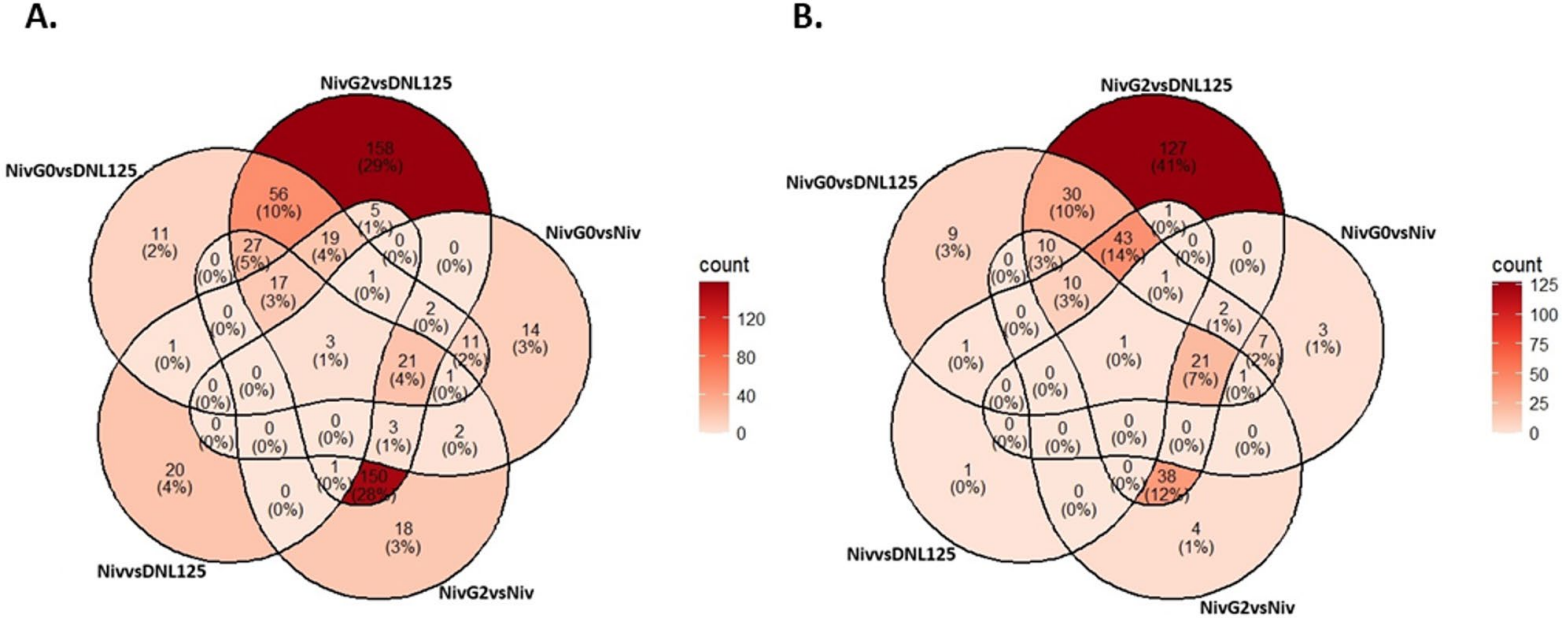
Venn-diagram of genes up-regulated in Nivolumab (Niv) producing glycomodified (G0 and G2) strains. Number of up-regulated (p-value < 0.01 and log2 fold change > 1) genes at days 2 (A) and 4 (B) of the bioreactor cultivations. Fold change was calculated against the samples of an empty parental strain (vsDNL125) or against the Nivolumab producing strain with native glycans (vsNiv)

In order to identify genes that were up-regulated because of the glycomodifications and not only due to mAb over-expression, Venn-diagrams were constructed to compare the number of genes up-regulated when fold changes were calculated against the empty parental strain along with those calculated against the Nivolumab producing, non-glycomodified strain (Fig. 5).

The 18 genes (day 2) that were up-regulated only in the G2 strain when compared to the Nivolumab strain with native glycans included GH7 cellobiohydrolase, kinesin-like protein, aminopeptidase, chitosanase, GT2 chitin synthase, DNA helicase and arginase. The 14 genes that were up-regulated (day 2) only in the G0 strain when compared to the Nivolumab strain with native glycans included GH7 endoglucanase, mitochondrial ATP synthase, potassium channel subunit, GH16 beta-glycosidase, GH55 beta-1,3-glucanase, MFS-type transporter, trichothecene 3-O-acetyltransferase, glutathione S-transferase, GH61 lytic polysaccharide monooxygenase, FAD-binding monooxygenase, acid phosphatase and glyoxylase.

**Fig. 6:**
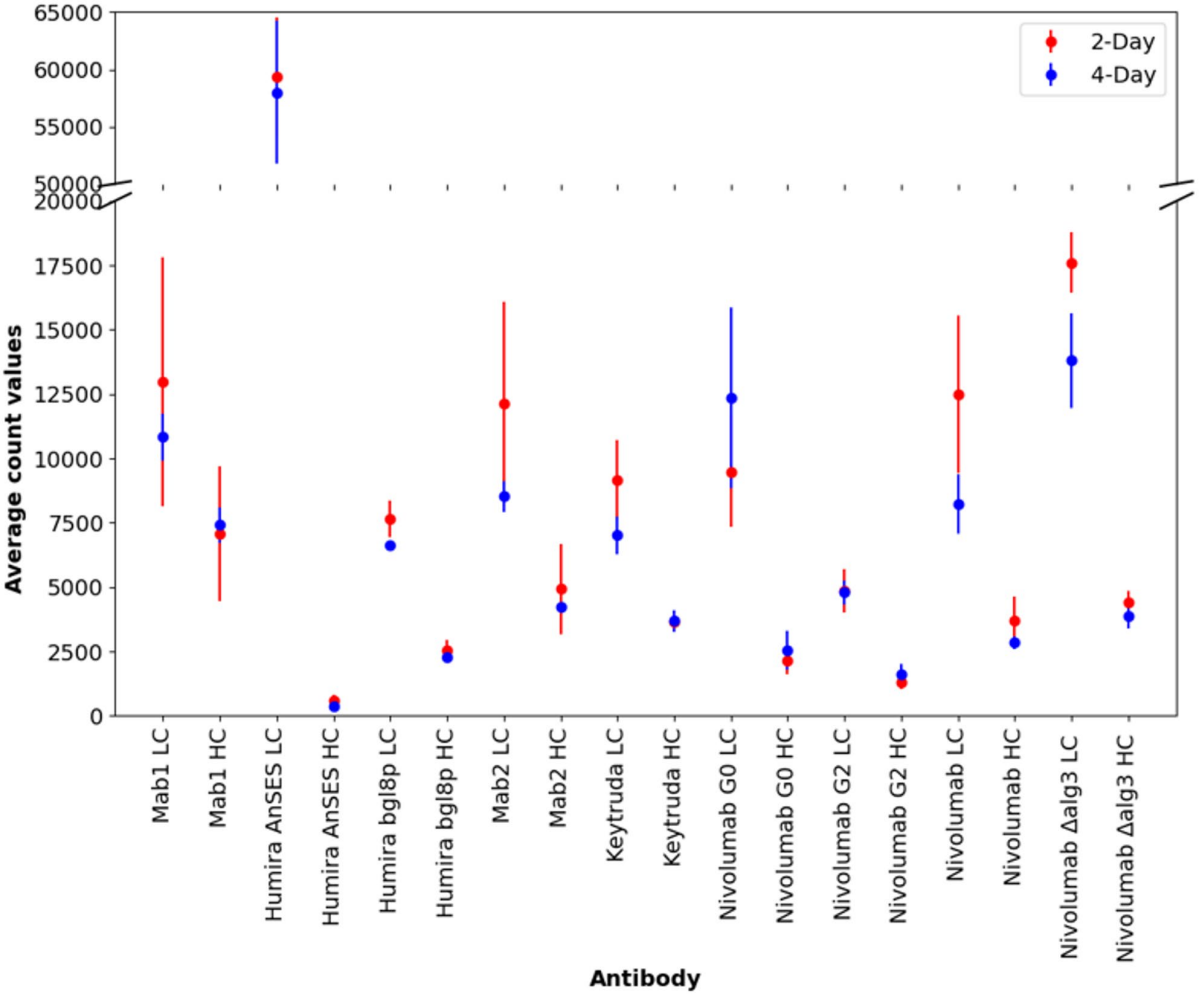
RNA-seq counts assigned to light chains and heavy chains of the over-expressed mAbs. Counts are the average from three biological replicate cultures. Standard deviations are shown

Most of the mAb heavy and light chain genes were expressed at very high levels (Fig. 6), but some differences were observed. When Humira was expressed under the AnSES promoter, LC expression was extremely high as compared to the HC level. The low expression of HC could possibly explain the overall low production of the antibody in this strain. This difference in gene expression levels was also reflected in the production levels of HC and LC based on Western blot analysis (Additional file 1., Supplementary Fig. 1). The heavy chain of mAb1 had the highest HC expression in the data, and LC expression was also high, which aligns with the high production of this mAb (Fig. 1). When comparing the expression in the glycomodified strains, Nivolumab LC expression was higher in the non-glycomodified strain and the Δalg3 strain compared to the G0 and G2 strains at day 2. At day 4, LC expression was higher in the G0 strain compared to the non-glycomodified strain. The expression of HC was lower than that of the LC in all the mAbs studied. As a comparison, the highest expressed C1 native genes had expression levels ranging from approximately 20 000 to 50 000, with one gene being > 100 000.

### Over-expression of folding and secretion related factors

The transcriptomics data generated from these cultures was used to select a number of genes for over-expression in C1. Differentially expressed genes with similar expression profiles were selected based on Mfuzz clustering. Clusters 4 and 5 contained secretion-related genes which were up-regulated in several antibody-producing strains (Fig. 2B). The genes of these two clusters were further annotated based on their homology to *Trichoderma reesei* genes, a blastx homology search against the SwissProt database, and InterPro domains in homologous proteins of Spoth2 in Joint Genome Institute´s MycoCosm fungal genomics portal. As a result, genes involved in ER entry, glycosylation, and folding, as well as in later stages of transport were identified. However, some exceptions were noted. For instance, up-regulation of secretion-related genes was weaker or missing in strains producing Humira mAb under the AnSES promoter or mAb1.

The expression of genes involved in ER entry was more consistently up-regulated in the Nivolumab-producing glycomodified G0 and G2 strains and the Keytruda strain compared to other strains. Upregulated genes involved in ER entry included signal peptidase subunits and translocation protein subunits. The highest number of genes involved in later transport events, from the ER to the Golgi and beyond, was up-regulated in the Nivolumab G2 strain. Genes involved in the synthesis of sugar donors and dolichol phosphate-linked precursors showed differential expression, especially in the Nivolumab G2 strain. Genes that participate in processing of glycan precursors together with oligosaccharyltransferase subunits were up-regulated especially in the Keytruda producing strain and in glycomodified Nivolumab strains.

All the genes selected from the transcriptome data for over-expression studies (Table 3) were identified from clusters 4 and 5. From the upregulated genes involved in protein folding, *pdi*-type gene, *sil1*, *scj1*, and *dnaJ* were selected for over-expression. Several homologs of the bacterial chaperone DNAJ, that cooperate with BIP, were identified. Consequently, an additional upregulated *dnaj* homologue was selected alongside *scj1*. These genes displayed higher expression levels in the glycomodified Nivolumab producing strains, particularly in the G2 strain (Additional file 1., Supplementary Fig. 3). Many of the genes also showed higher expression levels in the strain producing Keytruda, which is considered to be the most challenging of these mAbs for C1. A gene encoding an ORM1-like protein, involved in the unfolded protein response and lipid homeostasis, was also selected from the transcriptome data. This gene was significantly up-regulated only in the G2 Nivolumab strain (Additional file 1., Supplementary Fig. 3). From genes involved in transport from the ER to the Golgi, plasma membrane, and other organelles, the *erv29* and *erv46* genes of the COPII complex and a P-type Ca^2+^ -ATPase gene *pmr1* were chosen. From the upregulated genes involved in glycosylation, a UDP-galactose transporter homologue with a role in UDP-galactose transport to the Golgi lumen (*hut1*-like) and a dolichol-phosphate mannosyltransferase subunit 1 (*dpm1*) gene homologues were selected. The gene coding for DPM1 was significantly up-regulated only in the G2 strain, whereas the UDP-galactose transporter gene was up-regulated in all other strains except for those producing mAb1 and Humira under the AnSES promoter (Additional file 1., Supplementary Fig. 3). Expression of the UDP-galactose transporter gene was highest in the Nivolumab G2 strain.

**Table 3:** Genes selected from the transcriptome data for over-expression in glycomodified C1

Over-expressed gene	Description	JGI ID
*orm1*	Involved in sphingolipid synthesis and unfolded protein response	2300113
*pdi*-type	Protein disulfide isomerase	2295433
*dnaJ*-type	Chaperone	2309125
*scj1*	Chaperone-binding protein	2302852
*sil1*	Nucleotide exchange factor for an unfolded protein response protein	2296861
UDP-galactose transporter *hut1*	Involved in N-glycosylation	2138963
DOI-mannose synthase *dpm1*	Dolichol-phosphate mannosyltransferase subunit 1, Involved in N-glycosylation	2299450
*erv29*	Protein localized to COPII-coated vesicles	2294791
*erv46*	Protein localized to COPII-coated vesicles	2295135
*pmr1*	ATPase transporting Ca2 + and Mn2 + into Golgi	2294817

Additional genes were selected based on literature (Table 4). These genes included C1 homologues of *ero1*, calnexin, *bet1*, *sar1*, *bmh2b*, *cup5*, *kin2*, *vamp* and *sso1*. For some genes, the human counterpart that naturally processes mAbs in B cells was over-expressed instead of the native C1 gene. These included human *bip*, calreticulin, *cypb*, *mzb1* and *hyou1*.

**Table 4:** Genes selected based on literature for over-expression in glycomodified C1

Over-expressed gene	Description	JGI/NCBI ID	Ref.
*vamp*	Vesicle associated membrane protein	2299040	[30, 31]
*cup5*	subunit of the vacuolar ATPase V0 domain	2314502	[32]
*bmh2b*	14-3-3 protein, involved in vesicle transport	2114395	[32]
*kin2*	Serine-threonine protein kinase regulating exocytosis	2306759	[32]
*sso1*	Plasma membrane t-SNARE	2302649	[33]
*ero1*	Thiol oxidase involved in protein folding in ER	2313331	[32, 34]
*bet1*	Golgi vesicular membrane trafficking protein	2312668	[35]
*sar1*	GTPase component of COPII vesicle coat	2079774	[36]
calnexin	ER chaperone	2295005	[37]
human *bip*	a major HSP70 chaperone in the ER	CAA61201.1	[34]
Human calreticulin	Ca2 + binding protein	NP_004334.1	[38]
human *cypb*	Peptidyl-prolyl cis-trans isomerase	NP_000933.1	[39]
human *mzb1*	BIP chaperone complex component, contributes to oxidative folding of Ig	NP_057543.2	[40]
human *hyou1*	HSP70 protein involved in protein folding and secretion in ER, BIP co-chaperone	XP_016872584.1	[39]

To determine whether controlled expression of the selected factors would enhance monoclonal antibody production by C1, over-expression cassettes were constructed and transformed into a glycomodified Nivolumab production strain producing G2 type of glycans. The over-expression strains were cultivated in bioreactors for 7 days. Nivolumab production during cultivation was analyzed using Western blots (Additional file 1., Supplementary Fig. 4).

**Fig. 7:**
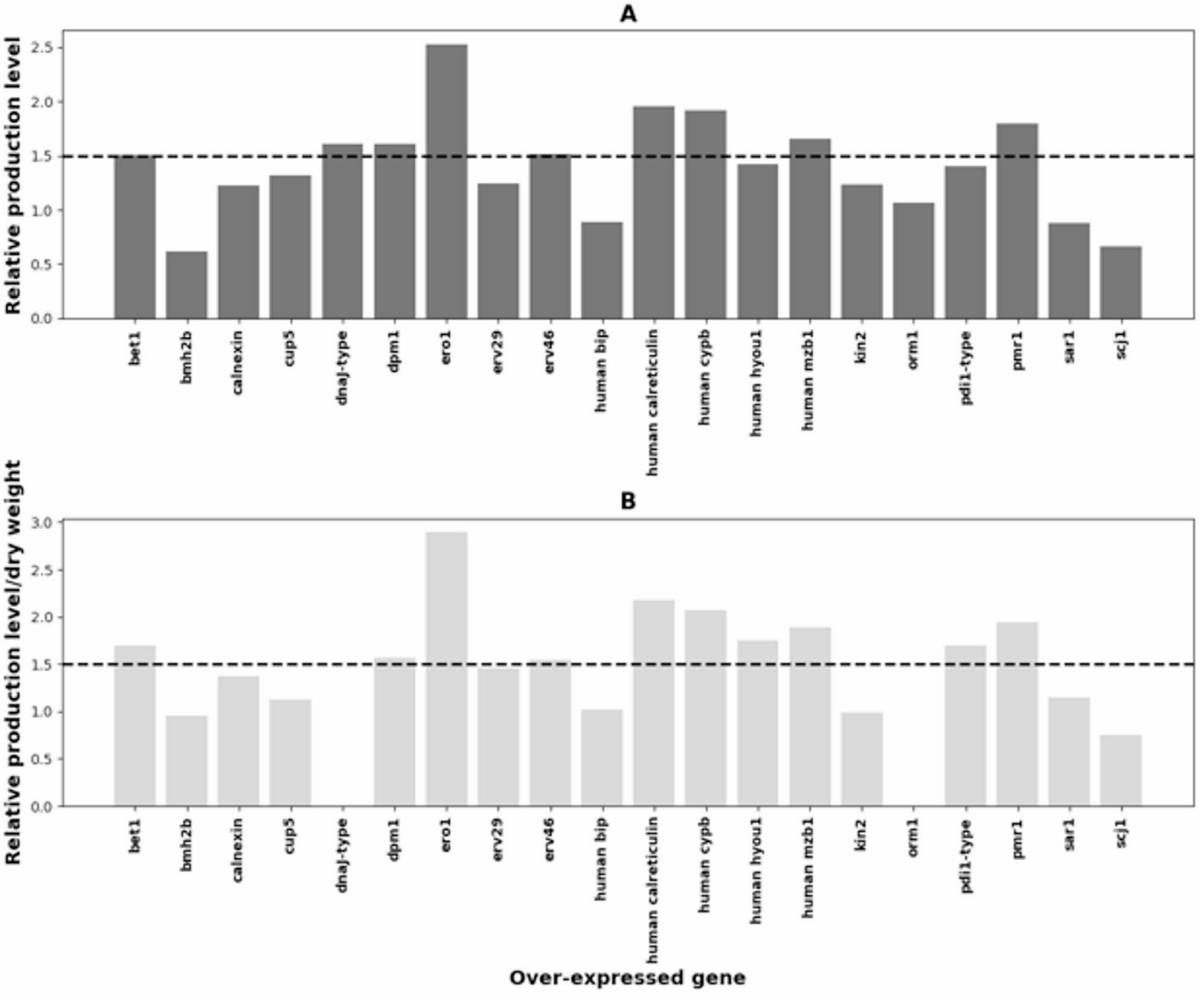
Normalized Nivolumab production levels of strains over-expressing genes encoding folding and secretion related factors. Production levels are calculated from 7-day bioreactor cultivations relative to the parental strain (**A**) and further normalized with the dry weight of the culture (**B**). Relative production levels of ≥ 1.5-fold were considered significant

Production levels were measured from 7-day samples by affinity purification with protein A columns and compared to the production level of the parental strain that was cultivated at the same time (Fig. 7A). Normalization of the production levels with dry weight values measured from the cultivations indicated that over-expression of the factors did not have a substantial negative effect on biomass production (Fig. 7B). Reliable dry weight values were not obtained from the cultivations of *orm1* and *dnaJ* over-expression strains due to technical errors and therefore they were omitted from the analysis. Volumetric values were used in selecting the factors that had a significant positive effect on mAb production. Because only one bioreactor cultivation was done for each over-expression strain and only one strain from each over-expressed gene was cultivated, fold changes of ≥ 1.5 were considered significant. Ten out of the 24 over-expressed genes had a positive effect on mAb secretion (Fig. 7A). The highest effects were obtained by over-expression of *ero1* and UDP-galactose transporter genes which increased production by 2.5- and 2.2-fold, respectively. Smaller positive effects were observed with *bet1*, *dnaj*, *dpm1*, *erv46*, human calreticulin, human *cypb*, human *mzb1* and *pmr1*, increasing mAb yield by 1.5-, 1.6-, 1.6-, 1.5-, 1.9-, 1.9-, 1.6- and 1.8-fold, respectively. Over-expression of human counterpart of the *hyou1* gene and *pdi1*-type gene resulted in 1.4-fold increase in Nivolumab production which is not considered significant due to lack of replicate bioreactor cultivations but suggests that these genes might also play a role in enhancing mAb production of C1. Effect of the over-expression to Nivolumab quality was assessed for selected strains using reducing and non-reducing Western blot and stained SDS PAGE analyses (Additional file 1., Supplementary Fig. 5).

## Discussion

Modifying the protein secretory pathway is an effective strategy for boosting protein production in fungi and for enhancing resistance to ER stress [34, 41]. For proper protein folding, the involvement of various chaperones and folding enzymes is needed. Among these, the transcription levels of BIP and protein disulfide isomerase (PDI) serve as indicators of cellular stress and the genes are frequently targeted for over-expression to enhance the yields of heterologous proteins [42–44]. BIP is the major hsp70 (heat shock protein 70 kD) protein of the ER lumen. PDI is a foldase that catalyses the formation and rearrangement of disulfide bridges during protein maturation in the ER [45]. Interplay of PDI and ER oxidoreductin (ERO1) is needed for disulfide bond formation [46]. In *Trichoderma reesei*, the expression of an IgG antibody Fab fragment led to an increase in the expression of the *pdi1* gene, suggesting that production of antibodies induces ER stress in filamentous fungi [47]. Over-expression of the *pdi1*, *ero1* and *bip1* genes has been demonstrated to enhance the secretion of a native β-glucosidase in *Trichoderma reesei* [34]. In *Pichia pastoris*, over-expression of the *pdi* gene led to a 1.9-fold increase in production of antibody Fab fragment [44].

In this study, genome-wide transcriptome analysis of C1 strains producing five different monoclonal antibodies, either with native glycans or human-like glycomodifications, was conducted to study the impact of mAb production and glycoengineering on gene expression and to identify genes involved in folding and secretion that could potentially present bottlenecks to the production of mAbs. Expression levels of heavy and light chain genes of the studied mAbs were among the highest in the whole transcriptome indicating that the bottleneck of mAb production is not in transcription or mRNA stability but instead cellular stress leads to induction of the folding and secretion genes particularly in the glycomodified strains and mAb strains displaying low production (for example the Keytruda producing strain). Furthermore, high secretory stress correlates with low antibody titer. The same effect has been detected with HEK293 cells, in which production of challenging mAbs resulted in the highest number of differentially expressed genes [48]. On the contrary, one of the best produced antibodies in our studies, mAb1, caused regulation of an extremely low number of genes. This can be considered surprising, as a full-length antibody is a rather complex molecule with four assembled chains and many disulfide bonds. In accordance, the level of heavy chain mRNA of mAb1 was the highest of the heavy chains. Production levels of over 24 g/l have been reached with mAb1 with another construct using a synthetic promoter in a non-glycomodified strain (data not shown). Nivolumab and mAb2 have also been produced at around 20–22 g/l levels in C1 with other strategies, and their production also showed a low number of changes in the transcriptome (Table 2).

When the two promoters of different strength were tested in expressing the Humira mAb, surprisingly the stronger promoter resulted in lowest production among all the mAb producing strains (Fig. 1). This was unexpected, especially considering the exceptionally high level of light chain mRNA in the same strain whereas the heavy chain mRNA was expressed at low level (Fig. 6). The reason for this imbalance between the heavy chain and light chain mRNAs is not known, but the low heavy chain mRNA level likely contributes to the low number of transcriptomic changes observed in this strain (Table 2). The extremely abundant LC mRNA might also compete out the HC mRNA from the translocation machinery, thus limiting heavy chain production. This kind of competition has been suggested to occur in the yeast *S. cerevisiae* during recombinant IgG production [39].

When comparing the Nivolumab Δalg3 and Nivolumab G0 strains to the Nivolumab strains with native glycans, many of the secretory pathway genes did not show significant up-regulation. However, significant regulation was observed when the Nivolumab G2 strain was compared to the non-glycomodified Nivolumab strain. This indicates that the Δalg3 and G0 modifications alone do not exceed the stress effect caused by mAb production. However, the additional stress caused by the G2 glycosylation machinery is sufficient to increase transcription beyond the levels induced by Nivolumab over-expression. This might indicate that the expression of galactosyltransferase GalT, within the C1 Golgi complex, causes strong secretion stress, or that the galactosylated glycans on the proteins hamper the function of the secretory pathway in some way. Genes that are uniquely up-regulated in the strains having G0 or G2 glycomodifications included several genes which might have a function in cell wall remodeling. It can be speculated that modification of the cell wall structure when the ER is overloaded may facilitate secretion. In the study of [49] secretion of proteins involved in cell wall loosening was up-regulated in an α-amylase over-production strain of *Aspergillus oryzae*. On the other hand, cell wall remodeling may also hinder secretion which is seen in the lower production level of the glycomodified strains.

To alleviate the pressure on the C1 secretion machinery during mAb production, 10 genes selected from the transcriptome data and 14 genes selected based on literature, were over-expressed individually in a Nivolumab producing C1 strain engineered to produce G2 glycans. Genes were selected from several important steps of the secretion pathway including initial folding, glycosylation, quality control and transport. From the upregulated genes involved in protein folding, a *pdi*-type gene together with *sil1*, *scj1* and *dnaJ* homologues were selected from the transcriptome data for over-expression. SIL1 is a nucleotide exchange factor for BIP [50, 51] and SCJ1, located in ER lumen where it interacts with BIP, is a homologue of the bacterial DNAJ chaperone [52]. Both SIL1 and SCJ1 function as co-chaperones that promote the ATPase cycle of BIP [53]. From these genes, over-expression of the *dnaJ* homologue resulted in the most significant increase (1.6-fold) in Nivolumab production. In contrast, *scj1* over-expression decreased production. The negative impact of *scj1* over-expression on heterologous protein production has been previously demonstrated in *Saccharomyces cerevisiae* [53, 54]. The deletion of *scj1* abolished heterologous laccase secretion in *S. cerevisiae*, indicating a delicate balance in achieving the optimal expression level of chaperone genes for enhanced production of heterologous proteins [54]. Chaperones participate in various cellular functions beyond protein folding, some of which may still be unknown. The effects observed when over-expressing chaperones are likely to reflect the influence on these diverse functions. The lectin-like chaperone calnexin was selected based on literature. Over-expression of calnexin has been shown to improve production of for example heterologous manganese peroxidase by *Aspergillus niger* [37]. Calnexin over-expression in C1 resulted in only 1.2-fold increase in Nivolumab production which was not considered significant.

Glycosylation has a positive impact on protein stability and secretion. However, modifying the glycosylation pathway to mimic that of humans can result in incorrect protein folding in the secretory pathway and the activation of UPR and ERAD pathways. The over-expression of a UDP-galactose transporter (resembling *hut1*) or a dolichol-phosphate mannosyltransferase subunit 1 (*dpm1*) gene of C1 increased Nivolumab production ~ 2.2- and 1.6-fold, respectively. Similarly, over-expression of the *S. cerevisiae dpm1* gene in *T. reesei* has been shown to increase protein secretion and the extent of protein glycosylation [55, 56]. In a recent study, the *hut1* gene was selected for over-expression in *S. cerevisiae* based on plasma cell differentiation transcriptomics data and was shown to have a small positive (1.4-fold) effect on antibody production [57]. The expression level of the C1 *hut1* gene was highest in the G2 Nivolumab strain. This, together with improvement of mAb production in the C1 G2 glycan producing strain, might indicate that the level of UDP-galactose in the Golgi complex could be limiting N-glycan galactosylation and protein secretion in this C1 strain.

From genes involved in transport from the ER to the Golgi, plasma membrane, and other organelles, the *erv29* and *erv46* genes, encoding coat protein complex II (COPII) components, as well as a P-type Ca^2+^ -ATPase *pmr1*, were selected for over-expression based on the transcriptome data. ERV29 is a transmembrane receptor protein localized to COPII-coated vesicles, playing a role in vesicle formation and the incorporation of specific secretory cargo [58]. In C1, over-expression of the *erv29* homologue had a positive effect on mAb production but did not reach 1.5-fold improvement. Over-expression of *erv29* in *S. cerevisiae* resulted in increased secretion of bacterial endoglucanase, a fungal β-glucosidase, and a short single-chain antibody fragment, when combined with other modifications to the secretory pathway [59]. In *A. niger*, deletion of ERV29 impairs glucoamylase secretion whereas over-expression has a slight but significant positive effect on glucoamylase production [60]. Co-expression of STE13 and ERV29 improved secretion of T4 lysozyme by *Yarrowia lipolytica* [61]. ERV46, in a complex with ERV41, identifies and retrieves escaped ER proteins, preventing their mislocalization and subsequent degradation in the vacuole [62]. Approximately 1.5-fold higher Nivolumab production, as compared to the parental strain, was achieved when the C1 *erv46* homologue was over-expressed. PMR1, located in the Golgi, is responsible for delivering Ca^2+^ and Mn^2+^ ions to the secretory pathway, where they are essential for various functions including transport, folding, glycosylation, degradation, and processing of secreted proteins [63–65]. Depletion of Ca^2+^ in the ER activates the UPR [18], and there are indications that Ca^2+^ is necessary for the interaction between PDI and calreticulin [66]. Over-expression of the C1 *pmr1* homologue resulted in a nearly two-fold increase in Nivolumab production in C1.

The SNARE genes *sso1* and *bet1* were selected for over-expression based on literature. SSO1 protein functions in the late stage of protein secretion by targeting and fusing the secretory vesicles derived from Golgi to the plasma membrane [67], whereas BET1 is involved in transport of the ER derived vesicles to the Golgi [35]. Over-expression of *sso1* or *bet1* in *S. cerevisiae* increased secretion of the β-glucosidase enzyme Cel3A from *Saccharomycopsis fibuligera* and cellobiohydrolase Cel7A from *Talaromyces emersonii*, respectively [33, 35]. In C1, *bet1* over-expression increased mAb production ~ 1.5-fold, while *sso1* over-expression showed a non-significant but positive effect (Fig. 7A). KIN2, a protein kinase involved in regulation of exocytosis [68], and BMH2, involved in the regulation of exocytosis and vesicle transport [69], were both selected based on a study showing that their over-expression in *Pichia pastoris* led to increase in secretion of a human antibody Fab fragment [32]. Also CUP5, a vacuolar ATPase subunit [70] and ERO1, the ER oxidoreductin, were selected based on the same publication in which an increase in antibody Fab fragment yield was detected in strains of *P. pastoris* over-expressing these genes [32]. Nivolumab production was increased 1.3-fold, 1.2-fold and 2.5-fold in *cup5*, *kin2* and *ero1* in C1 over-expression strains, respectively. On the contrary, *bmh2* over-expression decreased Nivolumab production, as did over-expression of the *sar1* gene, whose product is a small GTP-binding protein involved in secretory vesicle transport [36].

In addition to over-expressing native C1 folding and secretion related factors to enhance heterologous mAb production we also tested human folding factors that naturally process mAbs in B cells. Over-expression of human *bip1* decreased mAb production. This was not totally unexpected since, for example, the study of [37] found that production of manganese peroxidase by *A. niger* was negatively affected by *bipA* over-expression. Over-expression of human calreticulin and human CYPB peptidyl-prolyl cis-trans isomerase increased Nivolumab production nearly 2-fold, whereas human co-chaperone MZB1 had a smaller effect, about 1.6-fold increase. Calreticulin is a calnexin-type chaperone, which does not contain a transmembrane anchor and is localized in the ER lumen. Calreticulins are not found in fungi, and therefore it is interesting that the human protein enhanced mAb production significantly in C1. Human ER hsp70 protein HYOU1 over-expression resulted in 1.4-fold increase to mAb production, which was not considered significant. In a recent study, knocking out the *hyou1* gene of HEK293 cells increased mAb production [48]. MZB1 is a luminal endoplasmic reticulum protein that controls Ca^2+^ homeostasis and regulates antibody secretion of B cells [40]. Immunoglobulin secretion was increased in B cells over-expressing the *mzb1* gene. Human cyclophilin B (CypB) is a peptidyl-prolyl cis-trans isomerase (PPIase), the activity of which is needed for immunoglobulin fold formation and tetramer assembly [71]. In the study by [39], information about up-regulation of PPIases during differentiation of an immune system´s B cells into antibody secreting plasma cells was utilized to enhance antibody secretion by *S. cerevisiae* and the rate of peptidyl-prolyl isomerization was suggested to be a key factor in improving antibody secretion by yeast.

In summary, the highest increase in secretion of monoclonal antibody from a glycoengineered C1 strain was achieved by over-expressing folding related genes and genes involved in glycosylation, highlighting these processes as major bottlenecks in the secretion pathway. Additionally, maintaining Ca^2+^ homeostasis appears to be crucial for efficient antibody secretion. However, addressing bottlenecks at one stage of the secretion pathway may introduce new rate limitations at other stages, creating new bottlenecks. Therefore, the next step in optimizing the secretion of heterologous proteins by C1 involves testing combinations of these individual factors. Production levels of up to 13 g/l have been achieved in the C1 system for monoclonal antibodies with human-like glycans through further optimization of expression strategies and glycoengineering (our unpublished data). In comparison, over 10 g/l has been reached for mAbs produced in CHO cells in a process that is typically twice as long as the C1 process [72]. This highlights C1 as a strong alternative platform for monoclonal antibody manufacturing.

## Conclusions

This study focused on improving monoclonal antibody (mAb) production in the filamentous fungus *Thermothelomyces heterothallica* C1. By analyzing transcriptome data, it was shown that expression of monoclonal antibodies with low yields or with engineered glycans, induced a stronger ER stress response as compared with highly produced mAbs with native glycans. Transcriptome data combined with literature searches identified 24 factors related to protein folding and secretion which were subsequently over-expressed in C1. This approach led to a significant increase in mAb production in C1 strain producing human-like N-glycans. The over-expression of *ero1* or UDP-galactose transporter genes resulted in the highest improvements, with up to a 2.5-fold increase in production. These findings highlight the potential of genetic engineering of the secretory pathway to enhance the efficiency of mAb production in C1, making it a viable option for industrial-scale production.

## Supplementary Information

Below is the link to the electronic supplementary material.

Additional file 1: Additional tables and figures to the article.

Additional file 2: RNA-sequencing data.

## Data Availability

All data generated or analyzed during this study are included in this published article [and its supplementary information files].

## Abbreviations

mAb: Monoclonal antibody
HC: Heavy chain
LC: Light chain
ERAD: Endoplasmic reticulum-associated degradation
UPR: Unfolded protein response
ER: Endoplasmic reticulum
HSP: Heat shock protein
BIP: Binding immunoglobulin protein
CBH1: Cellobiohydrolase 1
GNT: Glucose N-acetyltransferase
MCB: Master cell bank
PMSF: Phenylmethanesulfonyl fluoride
RNA-seq: RNA sequencing
PCA: Principal component analysis
PDI: Protein disulfide isomerase
ERO: ER oxidoreductin
COPII: Coat protein complex II
PPIase: Peptidyl-prolyl cis-trans isomerase
