# Supplementary material for: Transcriptomics analysis identifies folding and secretion related genes for improving monoclonal antibody production in Thermothelomyces heterothallica C1
Source: Microb Cell Fact. 2026 Apr 2;25:124. doi: 10.1186/s12934-026-02989-w (PMC13170295; doi:10.1186/s12934-026-02989-w)
Supplement: Supplementary file 2 — Additional file 2: RNA-sequencing data. [file 12934_2026_2989_MOESM2_ESM.docx]

**Supplementary Table 1.** Oligonucleotide primers for screening transformants with correct integration to the *cbh1* or *alp6* locus and the absence of the *cbh1*/*alp6* coding region.

| Primer | Sequence | Target |
| --- | --- | --- |
| oMYT0087_bgl_pr_s4 | GACTCTGAACGAGCCGGG | *bgl8* promoter |
| oMYT0127_cbh1_5int_1 | ATCAGACCACGACGGGAC | 5´ region outside of *cbh1* locus |
| oMYT0129 | TAGCGCGAATACTGCTGTGG | 3´ region outside of *cbh1* locus |
| oMYT0157_cbh1_orf_for | GCTGACGCGAATGACACAG | *cbh1* open reading frame |
| oMYT0158_cbh1_orf_rev | CATGCCCTTGCCGTAGAAG | *cbh1* open reading frame |
| oMYT3157_TEF1p_rev | AAGTCCACATTCTGCAGCAAC | *tef1* promoter |
| oMYT306 | TCGTCTACGTTCCAACAGCC | 5´ region outside of *alp6* locus |
| oMYT3158_pyr4_f | GTCAGTCTTGTGCGTGTCCTG | *pyr4* marker |
| oMYT0307 | ACATCCCTGATGCACAGTGG | 3´ region outside of *alp6* locus |
| oMYT1992_alp6-del-F1 | CTACATCGATCACGGACACG | *alp6* open reading frame |
| oMYT1993_alp6-del-R1 | GCAGAAGTCCTTGGAGATGC | *alp6* open reading frame |

A.


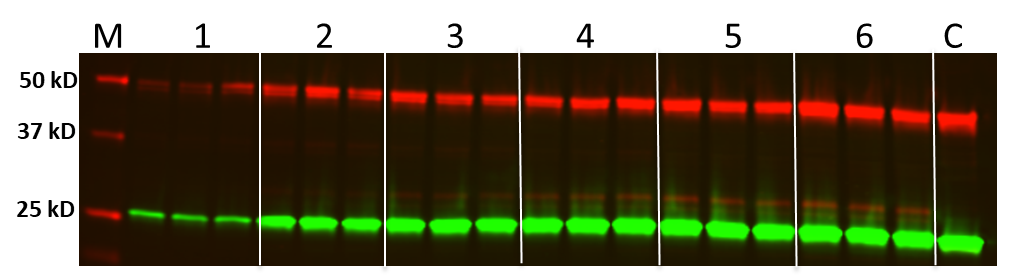


B.


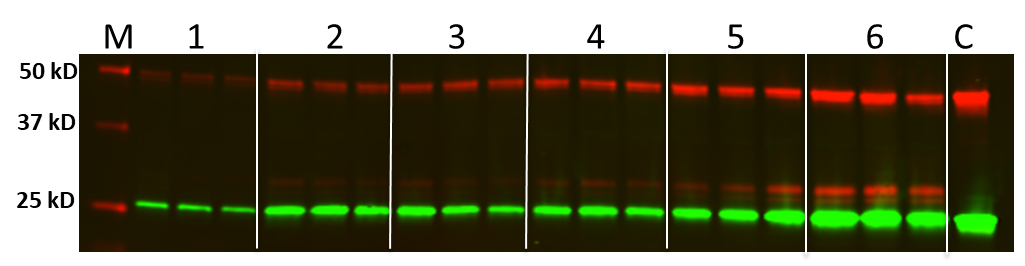


C.


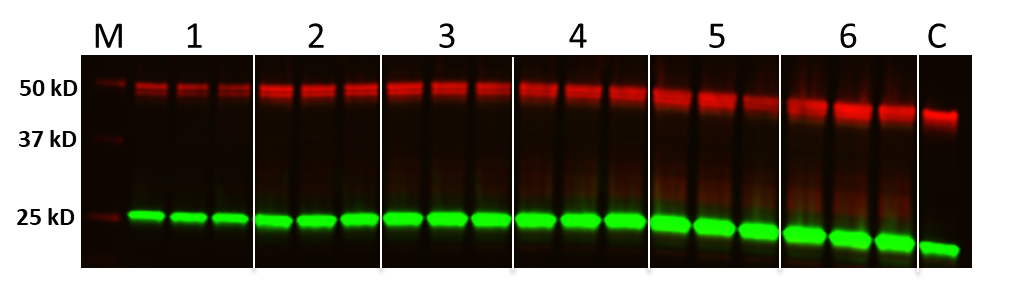


D.


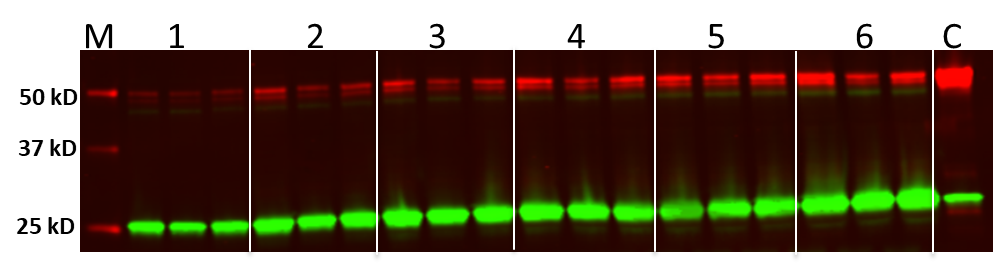


E.


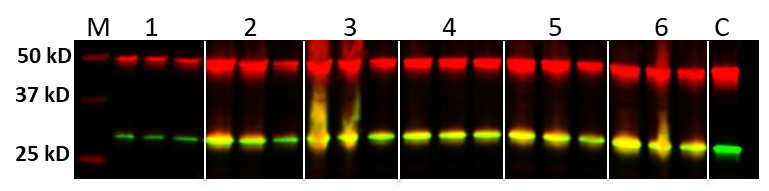


F.


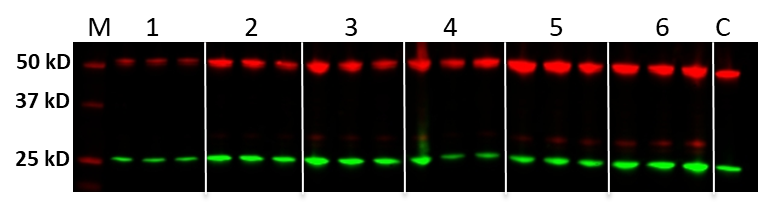


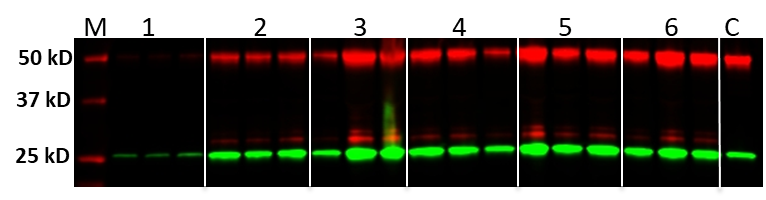


H.

G.


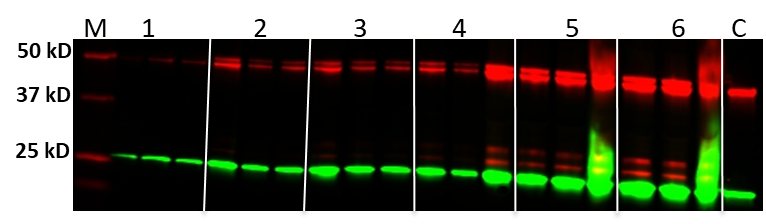


I.


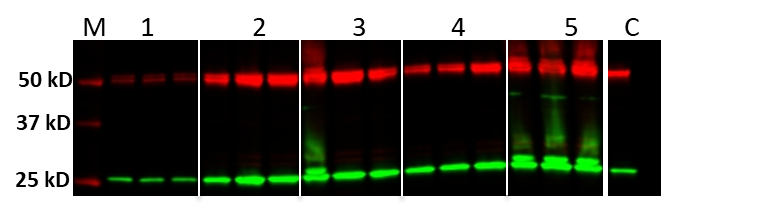


**Supplementary Figure 1.** Western blot analysis of supernatant samples from bioreactor cultivation of strains expressing different monoclonal antibodies. Samples of three biological replicates from cultivation time points 1-5/6 days were analysed. A. mAb2 strains and 400 ng of mAb2 as a control. B. Keytruda strains and 400 ng of mAb2 as a control. C. Humira (bgl8p) strains and 200 ng of Humira as a control. D. Humira (AnSESp) strains and 200 ng of Humira as a control. E. mAb1 strains and 400 ng of mAb1 as a control. F. Nivolumab/native glycan strains and 400 ng of mAb2 as a control. G. Nivolumab/G0 strains and 400 ng of mAb2 as a control. H. Nivolumab/G2 strains and 400 ng of mAb2 as a control. I. Nivolumab/Δalg3 strains and 400 ng of mAb2 as a control. The yellow light chain band in the mAb1 Western in (E) is caused by the fact that in this strain background a proteolytic fragment derived from the heavy chain co-migrates with the light strain (the merge of the red and green labels is yellow). For HC detection, anti-human IgG F(c) Goat Polyclonal Antibody DyLight680 (Li-Cor) was used. For kappa LC detection, Goat anti-human Kappa Light Chain secondary antibody DyLight 800 (Li-Cor) was used. For lambda LC detection, anti-human lambda light chain antibody (abcam) was used together with goat anti-mouse 800CW


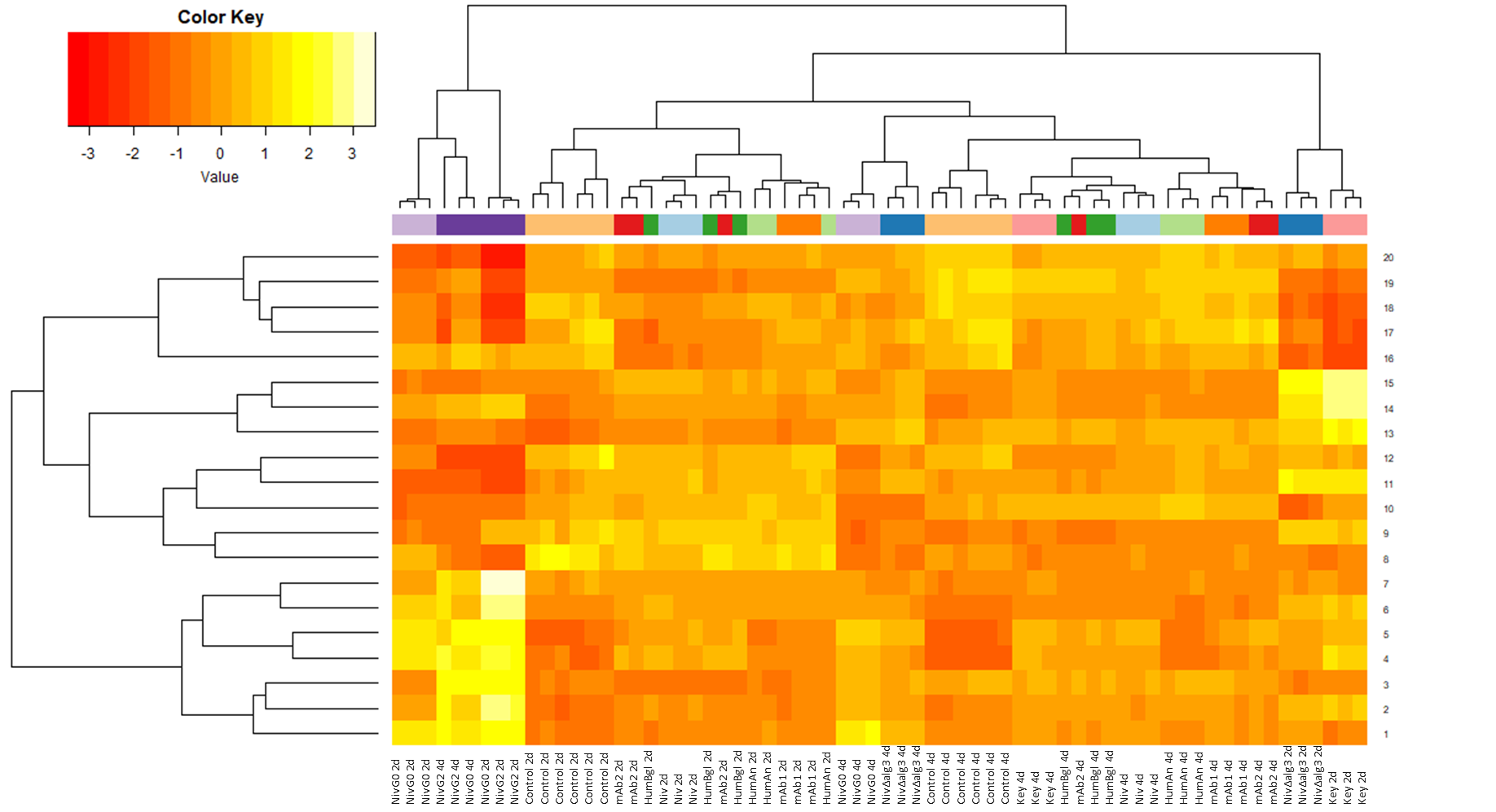


**Supplementary Figure 2.** Heatmap visualizing the differences in expression profiles based on Mfuzz clustering of C1 transcripts from different mAb production strains and parental strains at two different bioreactor cultivation time points. Columns represent the culture conditions (strain and time point) and rows the profiles of cluster centers. Cluster numbering is shown (right). Color key illustrates the expression changes from Mfuzz analysis.


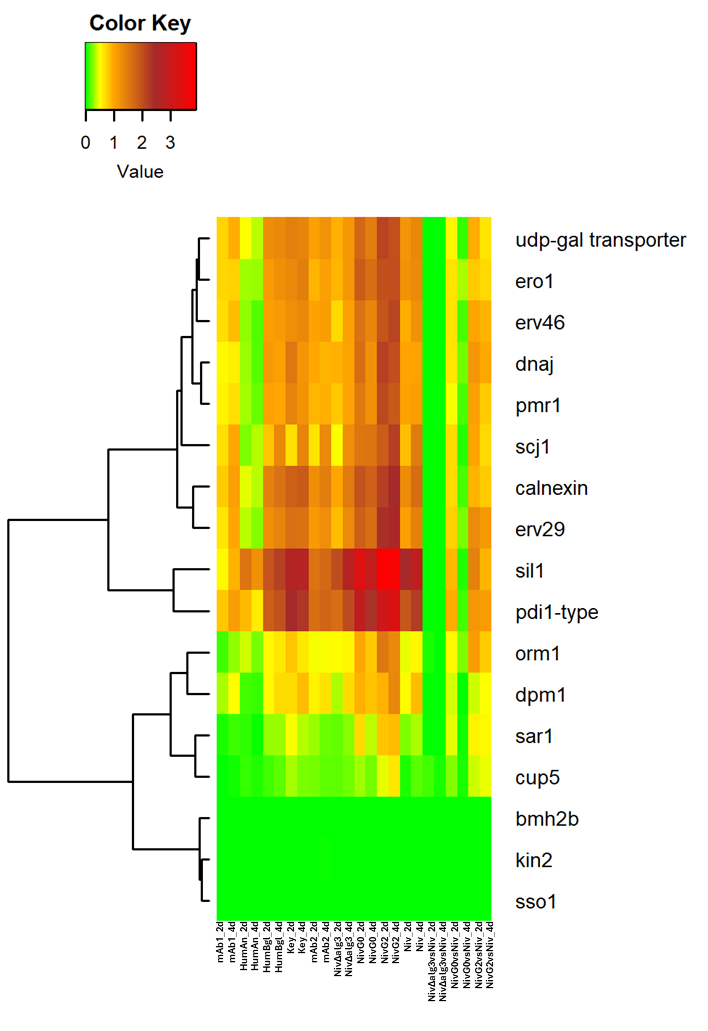


**Supplementary Figure 3.** Heatmap visualizing fold changes of C1 transcripts encoding different genes of secretory pathway. Columns represent the culture conditions (strain and time point) and rows the fold changes. Fold change was calculated against the samples of an empty parental strain or against the Nivolumab producing strain with native glycans (vsNiv). Color key illustrates the fold changes.

A.


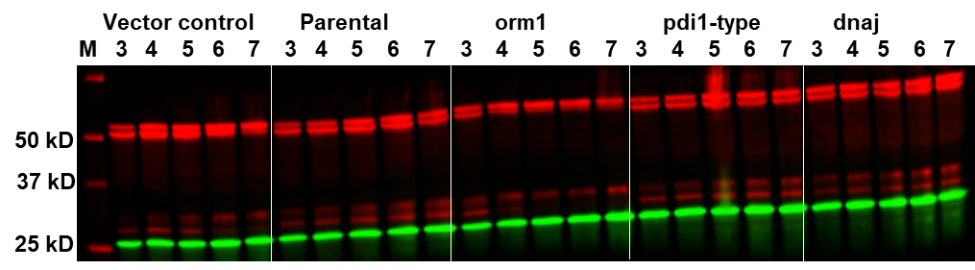


B.


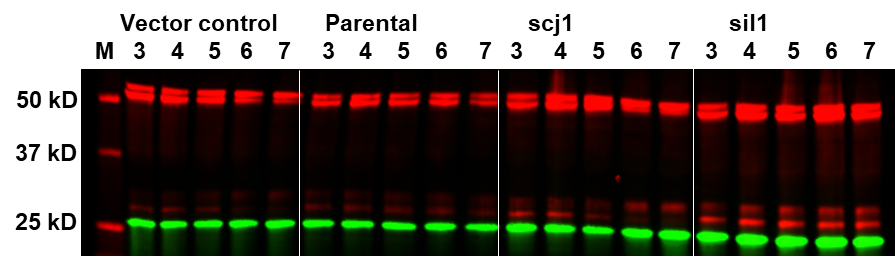


C.


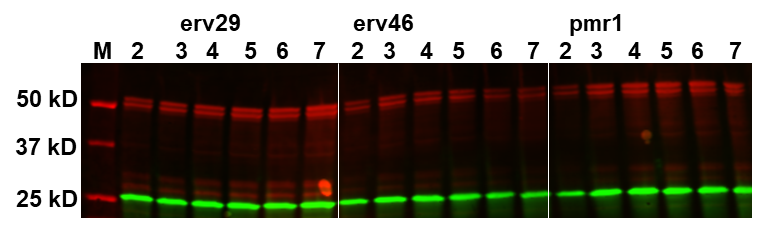


D.


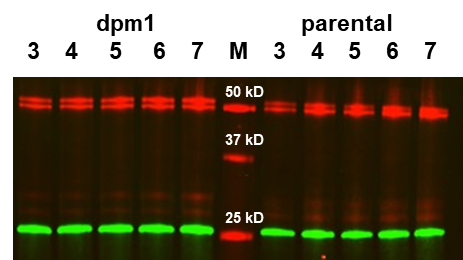


E.


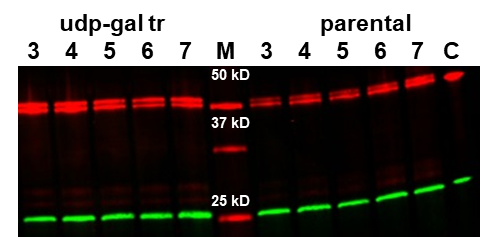


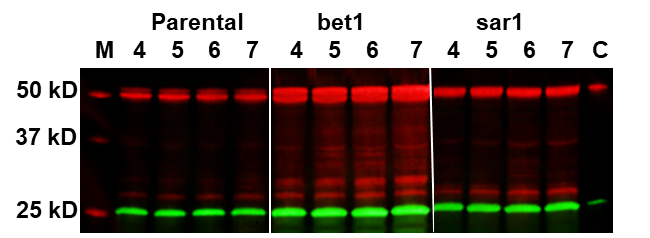


F.

G.


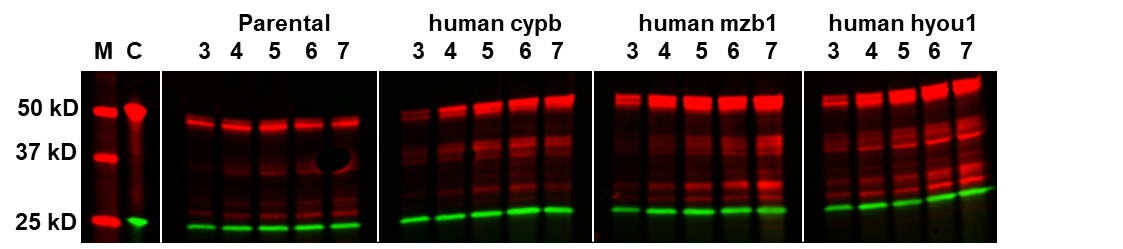


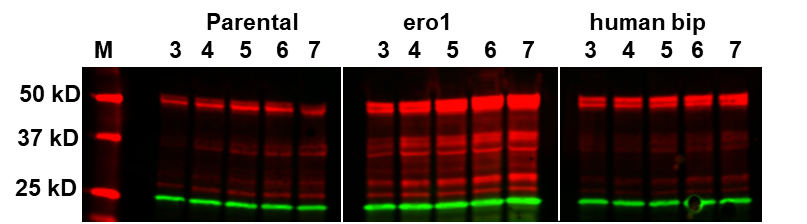


I.

H.


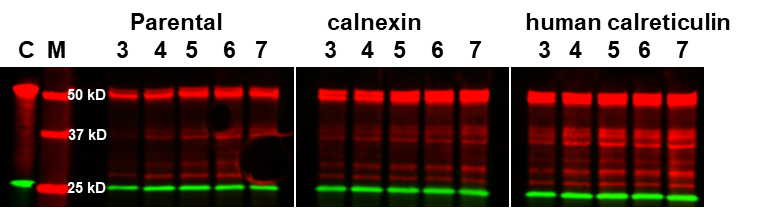


J.


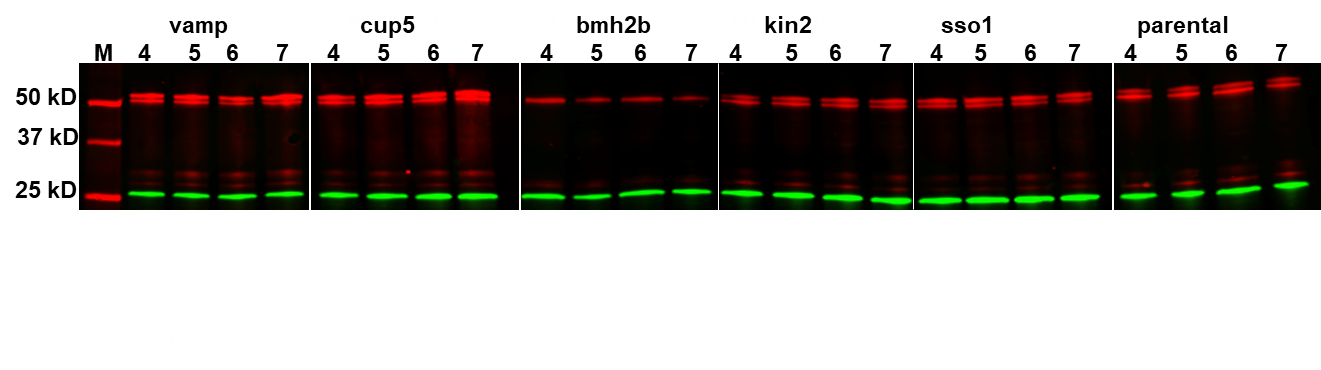


**Supplementary Figure 4.** Western blot analysis of supernatant samples from bioreactor cultivation of Nivolumab producing glycomodified C1 strains over-expressing different factors involved in folding and secretion of proteins. The numbers above the panels correspond to time points (in days) of the cultures. A. over-expression of *orm1*, *pdi1* and *dnaj* genes. B. over-expression of *scj1* and *sil1* genes. C. over-expression of *erv29*, *erv46* and *pmr1* genes. D. over-expression of *dpm1* gene. E. over-expression of UDP galactose-transporter gene. F. over-expression of *bet1* and *sar1* genes. G. over-expression of human *cypb*, human *mzb1* and human *hyou1* genes. H. over-expression of *ero1* and human *bip* genes. I. over-expression of C1 calnexin and human calreticulin genes. J. over-expression of *vamp, cup5, bmh2b, kin2* and *sso1* genes. Parental strain M4575 and vector control strain (M4575 with empty expression vector integrated to *alp6* locus) were included in the analysis as controls. Commercial Opdivo or mAb2 were used as control protein. The double band detected for the heavy chain corresponds to a glycosylated and non-glycosylated form, as a relatively low N-glycan site occupancy is detected in this strain. For HC detection, anti-human IgG F(c) Goat Polyclonal Antibody DyLight680 (Li-Cor) was used. For LC detection, Goat anti-human Kappa Light Chain secondary antibody DyLight 800 (Li-Cor) was used.


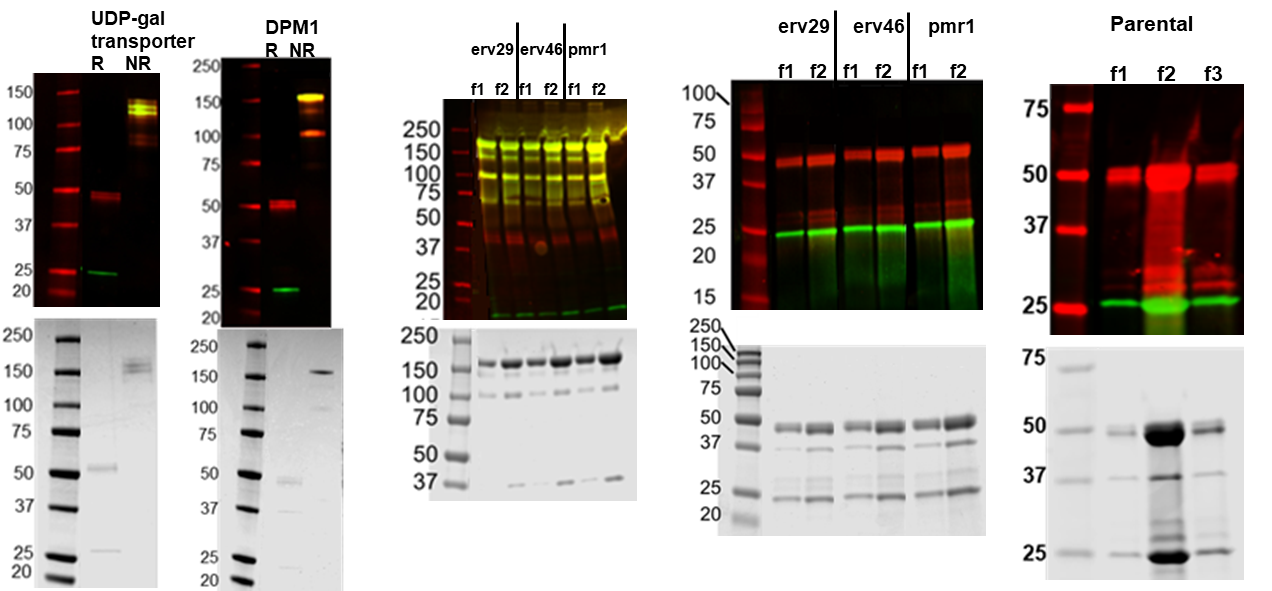


**D.**

**C.**

**E.**

**B.**

**A.**

**Supplementary figure 5.** Western blot and stained SDS PAGE gel analyses of Nivolumab preparations purified by protein A chromatography. Nivolumab was purified from bioreactor cultivations of strains over-expressing UDP-galactose transporter (A), *dpm1* (B), *erv29*, *erv46* and *pmr1* (C-D) and from the parental strain cultivation (E). Figures A and B include both reduced (R) and non-reduced (NR) samples. Fractions (f) in figure C are all non-reduced whereas figures D and E include only reduced samples. For HC detection, anti-human IgG F(c) Goat Polyclonal Antibody DyLight680 (Li-Cor) was used. For LC detection, Goat anti-human Kappa Light Chain secondary antibody DyLight 800 (Li-Cor) was used. Size (kD) of the molecular weight marker is marked to the left side of the figures.


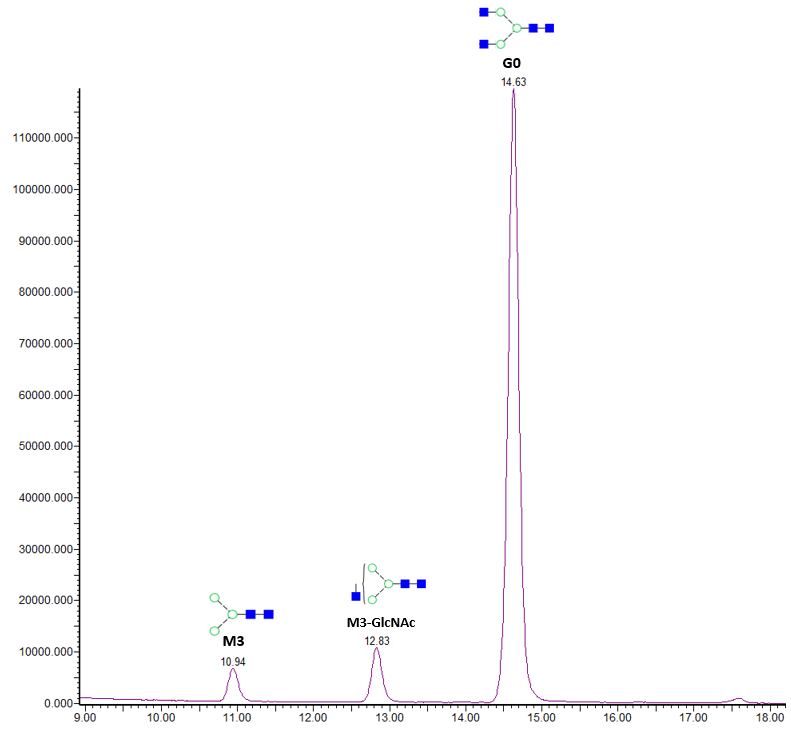


A.


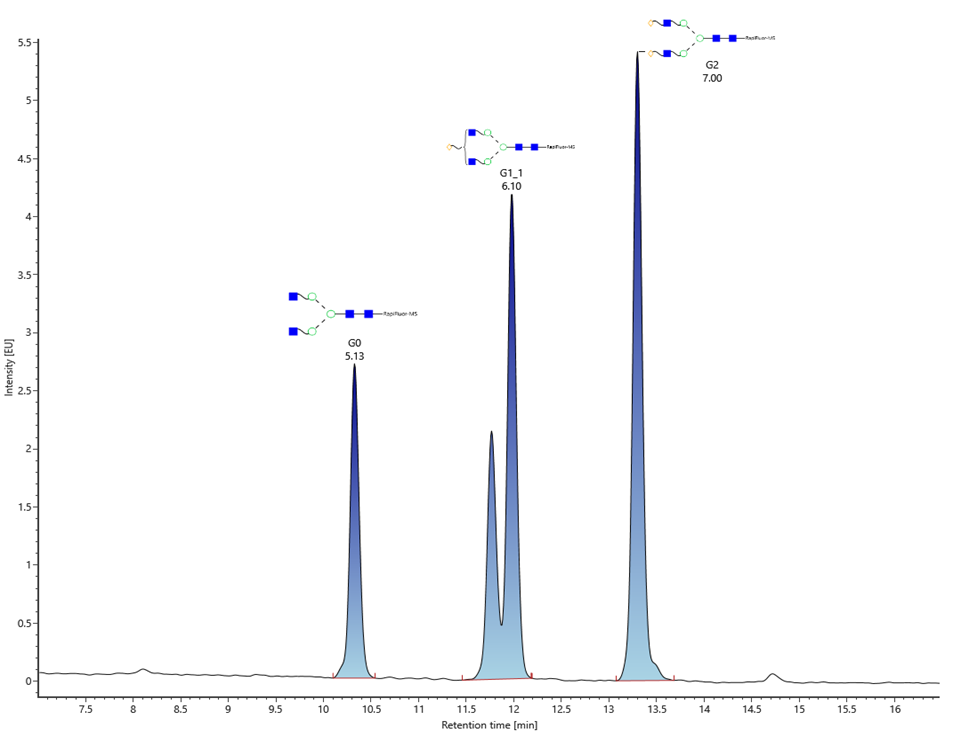


B.

**Supplementary Figure 6.** The N-glycan patterns of the glycoengineered C1 strains producing Nivolumab with G0 (A) and G1/G2 (B) glycans. Different glycans detected are marked to the figure together with their retention times.
